# The mediating role of DNA methylation clocks in associations of race, ethnicity, education, income, and occupation with mortality: findings from NHANES 1999-2002

**DOI:** 10.18632/aging.206377

**Published:** 2026-05-08

**Authors:** Hanyang Shen, Nicole Gladish, Tiffany Fan, Andres Cardenas, Belinda L. Needham, David H. Rehkopf

**Affiliations:** 1 Department of Epidemiology and Population Health, Stanford University, Palo Alto, CA 94305, USA; 2 Institute for Computational and Mathematical Engineering, Stanford University, Palo Alto, CA 94305, USA; 3 Department of Pediatrics, Stanford University, Palo Alto, CA 94305, USA; 4 Department of Epidemiology, University of Michigan, Ann Arbor, MI 48109, USA; 5 Department of Health Policy, Stanford University, Palo Alto, CA 94305, USA; 6 Department of Medicine (Primary Care and Population Health), Stanford University, Palo Alto, CA 94305, USA; 7 Department of Sociology, Stanford University, Palo Alto, CA 94305, USA

**Keywords:** race and ethnicity, social position, epigenetic aging, mediation analysis, mortality disparities

## Abstract

Race, ethnicity, education, income, and occupation are important social factors affecting health and longevity. DNA methylation aging biomarkers may mediate the effects of these social factors on mortality, but so far, no studies have investigated the extent of this mediation systematically in a nationally represents samples of the U.S. population. This study aims to provide systematic evidence on the mediating proportion of DNA methylation clocks between these social factors and mortality using the US National Health and Nutrition Examination Survey (NHANES) 1999-2002 (N=2,402) linked with National Death Index mortality data through 2019. After adjusting for gender, age, age2, and nativity, multiple DNA methylation clocks significantly mediated the pathways between social factors and all-cause mortality. Among all the 13 DNA methylation biomarkers available in NHANES, GrimAge2 consistently exhibited the strongest positive mediation capturing the social disparities on mortality up to 52% (95%CI: 26%-128%), followed by the DunedinPoAm. These effects generally exceeded the mediation proportion of traditional clinical risk factors collected in our study (such as C-reactive protein). This study suggested that DNA methylation epigenetic biomarkers may have utility as clinical aging indicators of the biological embedding of social disadvantage.

## INTRODUCTION

For most documented contexts and time periods, there is a strong association between lower socioeconomic position and risk of higher mortality [1, 2]. The theory of social stratification posits that social stratification caused by a combination of factors, particularly race, ethnicity, and socioeconomic position, would influence health outcomes through differential access to resources, power, and opportunities [3, 4]. These adverse effects even can undermine the beneficial effects from other social exposures such as social cohesion and social resistance [5]. These health disparities are reflected in key social stratification factors such as race and ethnicity, educational attainment, income, and occupation. Studies report notable differences in life expectancy across these dimensions. For instance, according to recent estimates, White Americans who reach age 15 have a life expectancy of 63 years, compared to 59 years for Black Americans and 66 years for Hispanic Americans [6]. Likewise, individuals with an income at or above 400% of the poverty threshold have a life expectancy of 60 years at age 18, while those living below the poverty line have just 49 years [7]. Similar disparities are also observed across different education levels and occupational groups [7–9].

To address these health inequalities, researchers have explored biological pathways to explain these embodiments from outset disadvantages to inner systems. One proposed mechanism is the early onset of chronic diseases due to behavioral and lifestyle factors, such as poor diet, smoking, and sedentary lifestyles [9]. Another mechanism involves environmental exposures, including local environment, household exposures, and occupational hazards, which disproportionately affect lower socioeconomic groups [10]. For example, individuals exposed to air pollution experience higher rates of cardiovascular diseases and hospitalizations, with these exposures being more prevalent in lower socioeconomic neighborhoods [11]. A third pathway suggests that chronic psychosocial stress resulting from economic instability, discrimination, and social deprivation leads to systemic inflammation and dysregulated glucose metabolism, both of which contribute to a higher risk of mortality [12].

Identifying reliable biomarkers that capture these pathways is critical for strengthening causal evidence linking distal social exposures early in the life to pathobiological disease processes occurring many years later. However, most traditional biomarkers reflect only a single biological process, limiting their ability to provide a comprehensive picture of health inequalities. For example, inflammation biomarkers have shown only modest mediation effects in explaining the relationship between socioeconomic status and mortality [13]. Moreover, social determinants of health and health policy changes often impact multiple biological and behavioral pathways differently, making it difficult to evaluate the correct direction of overall health impact using a single biomarker. For instance, a study on Earned Income Tax Credit found that this policy affected various health outcomes in different ways—some beneficial and others potentially detrimental [14]. Developing biomarkers that integrate signals across multiple pathways could therefore offer more holistic insights. Such integrative biomarkers would be especially useful for assessing the short-term health effects of social policy changes, such as education reforms, housing programs, tax deductions, and environmental regulations.

DNA methylation clocks offer a potential solution as they may function in some cases as comprehensive biomarkers that capture cumulative biological effects of social and environmental exposures. Epigenetic mechanisms regulate gene expression by modifying DNA without altering the genetic sequence. DNA methylation, in which methyl groups are covalently bound to cytosine bases, influences gene accessibility and expression and has been associated with disease risk and mortality [15, 16]. DNA methylation clocks, which estimate either chronological age or physiological aging based on DNA methylation patterns, have been shown to be significantly associated with race and ethnicity, educational attainment, income, and occupation [17–23]. In addition, some of these clocks also strongly correlate with mortality and morbidity outcomes by design. However, only a limited number of studies have examined whether DNA methylation clocks mediate the relationships between race, ethnicity, socioeconomic positions and mortality disparities [24–27]. Existing evidence suggests that epigenetic aging biomarkers, especially the DNA methylation biomarkers trained on physiological measures, can significantly mediate these associations across diverse cohorts, yet previous studies have not simultaneously compared multiple social exposures or multiple mediators representing different biological pathways within a unified analytical framework.

To address this gap, the present study leverages nationally representative data from National Health and Nutrition Examination Survey (NHANES) to evaluate the mediating role of thirteen DNA methylation clocks trained for different purposes. These clocks are compared with traditional clinical biomarkers to assess their relative contributions to disparities caused by social stratification factors in mortality. The findings provide novel insights into the biological embedding of social disadvantages and highlight the potential utility of epigenetic aging measures for advancing research on the health impacts of social determinants.

## RESULTS

### Descriptive characteristics and survival analysis

The unweighted sample statistics for this study sample are presented in Table 1. Among the 2,402 participants in the analysis, 947 deaths (39%) were observed up to Dec 31, 2019. Of these, 164 were attributed to cardiovascular-related causes, while 197 were due to cancer. The racial and ethnic composition of the sample included 39% White Americans, 22% Black Americans, 36% Hispanic, and 3.5% individuals identifying as another race or mixed race. In terms of socioeconomic characteristics, 46% of participants had not completed high school, 38% had household income between two and five times of the poverty thresholds, and 42% reported their occupation as semi-routine blue-collar. The sample was 49% female and 25% foreign-born. The age of the participants ranged from 50 to 84 years, with the mean age as 65 years (SD=9).

**Table 1:** Descriptive statistics for socioeconomic positions, demographic covariates, and clinical measures, NHANES 1999-2002 (n=2,402).

	**Overall participants (N=2,402)**			**Deceased participants (N=947)**			**Undeceased participants (N=1,455)**	
**Categorical Variable**	**Count**	**(Percentage)**		**Count**	**(Percentage)**		**Count**	**(Percentage)**
Race and ethnicity								
Non-Hispanic White	943	(39.3%)		399	(42.1%)		544	(37.4%)
Non-Hispanic Black	516	(21.5%)		225	(23.8%)		291	(20.0%)
Hispanic	860	(35.8%)		300	(31.7%)		560	(38.5%)
Another race or ethnicity	83	(3.5%)		23	(2.4%)		60	(4.1%)
Educational attainment								
College degree or higher	370	(15.4%)		96	(10.1%)		274	(18.8%)
Some college or associates degree	440	(18.3%)		137	(14.5%)		303	(20.8%)
High school/GED	496	(20.7%)		201	(21.2%)		295	(20.3%)
Less than high school	1096	(45.6%)		513	(54.2%)		583	(40.1%)
Family income								
PIR 5+	399	(16.6%)		87	(9.2%)		312	(21.4%)
PIR 2-5	923	(38.4%)		337	(35.6%)		586	(40.3%)
PIR 1-2	707	(29.4%)		339	(35.8%)		368	(25.3%)
PIR<1	373	(15.5%)		184	(19.4%)		189	(13.0%)
Longest held occupation								
White collar and professional	535	(22.3%)		178	(18.8%)		357	(24.5%)
White collar, semi-routine	411	(17.1%)		154	(16.3%)		257	(17.7%)
Blue collar, high skill	389	(16.2%)		173	(18.3%)		216	(14.8%)
Blue collar, semi-routine	1004	(41.8%)		404	(42.7%)		600	(41.2%)
Never worked	63	(2.6%)		38	(4.0%)		25	(1.7%)
Gender								
Women	1172	(48.8%)		406	(42.9%)		766	(52.6%)
Men	1230	(51.2%)		541	(57.1%)		689	(47.4%)
Nativity								
US born	1794	(74.7%)		773	(81.6%)		1021	(70.2%)
Foreign born	608	(25.3%)		174	(18.4%)		434	(29.8%)
Physical activity								
Active	1171	(48.8%)		375	(39.6%)		796	(54.7%)
Sedentary	1231	(51.2%)		572	(60.4%)		659	(45.3%)
Alcohol consumption								
Abstainer	1033	(43.0%)		466	(49.2%)		567	(39.0%)
Alcohol drinker	1369	(57.0%)		481	(50.8%)		888	(61.0%)
	**Mean**	**(SE)**		**Mean**	**(SE)**		**Mean**	**(SE)**
Age	65.11	(9.31)		70.41	(8.77)		61.66	(7.93)
Healthy eating index	53.48	(12.86)		52.82	(12.86)		53.91	(12.84)
Smoking pack years	15.44	(24.53)		20.41	(28.75)		12.20	(20.72)
Waist to thigh ratio	1.96	(0.21)		2.02	(0.20)		1.92	(0.20)
BMI	28.82	(5.76)		28.38	(6.01)		29.11	(5.58)
Total cholesterol (mmol/L)	5.45	(1.06)		5.40	(1.14)		5.48	(1.00)
HDL-cholesterol (mg/dL)	51.83	(16.08)		51.59	(16.96)		51.98	(15.48)
LDL-cholesterol (mg/dL)	128.42	(31.60)		126.51	(32.62)		129.67	(30.87)
Glucose	107.22	(45.09)		114.47	(57.01)		102.51	(34.44)
C-reactive protein	0.60	(1.24)		0.70	(1.28)		0.53	(1.20)

The correlations between all the mediators we examined are shown in Figure 1. Most clocks had strong correlations with chronological age (r>0.7), with somewhat more moderate correlations for Weidner and Vidal-Bralo (r=0.5 and r=0.6, respectively), and Horvath predicted telomere showed moderate negative association with chronological age as the correlation coefficient was -0.6. Only DunedinPoAm and Yang epigenetic age showed weaker correlations with chronological age, r=0.04 and r=0.2, respectively.

**Figure 1:**
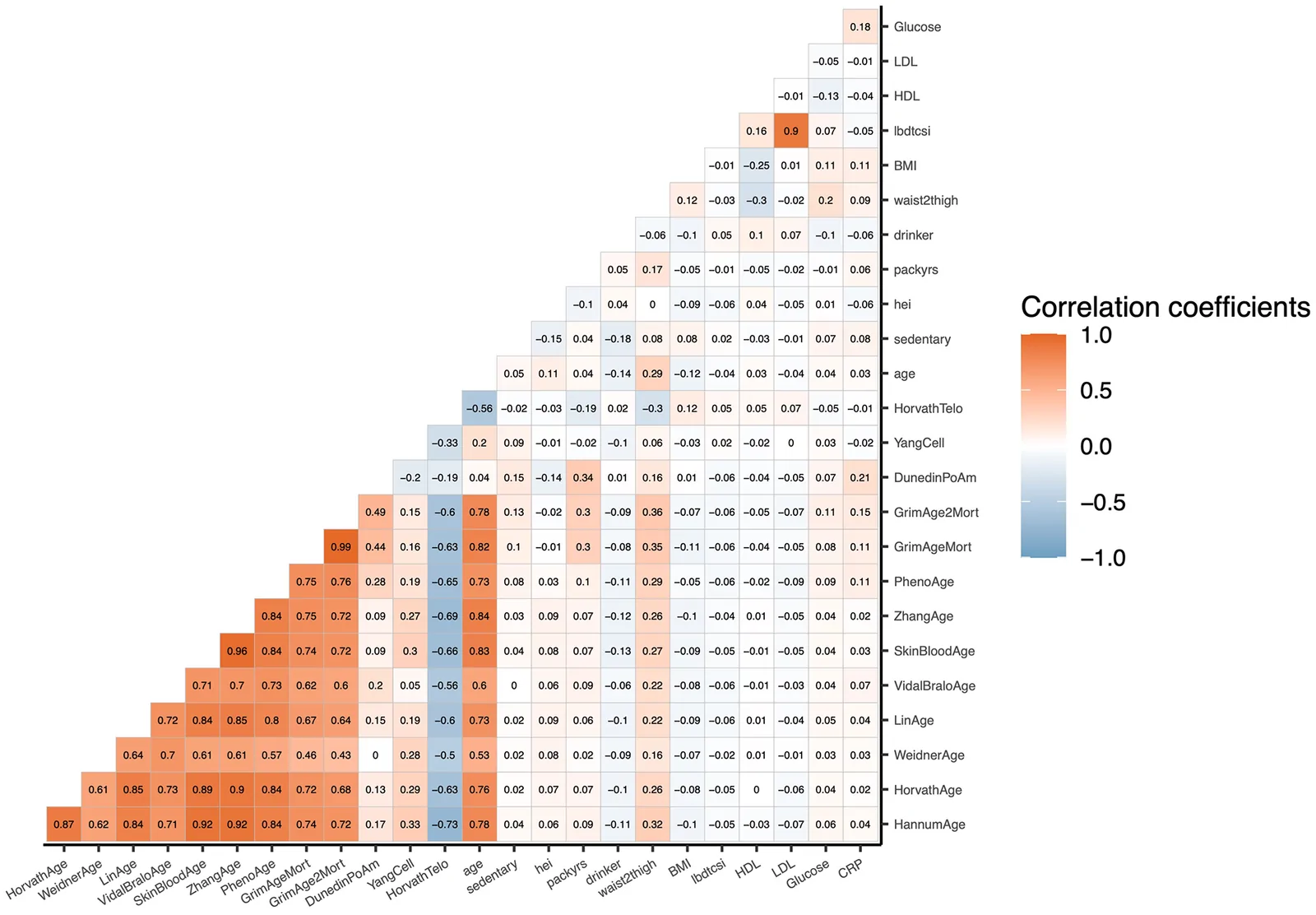
**Raw correlation matrix of potential mediators and age in NHANES 1999–2002 (n = 2,402).** Mediators include chronological age, physiological functioning clocks, pace-of-aging clock, clocks trained on other aging biomarkers, as well as behavioral, anthropometric, and clinical variables.

The estimated coefficients from the survival analysis models (without any mediators) are presented in Supplementary Table 1. Compared to White participants, Black and Hispanic participants exhibited significantly higher risks of all-cause mortality, with hazard ratios (HR) of 1.51 (95%CI: 1.25-1.82) and 1.40 (95%CI: 1.12-1.75), respectively, after adjusting for gender, age, age^2^, and nativity. All categories of lower educational attainment and lower household income were associated significantly with higher risks on all-cause mortality compared to the most advantaged groups, with HRs ranging from 1.63 (95%CI: 1.24-2.14) to 3.00 (95%CI: 2.18-4.12) with the most disadvantaged groups showing the biggest ratios for mortality. Individuals in high-skilled blue-collar and semi-routine blue-collar occupations showed significantly higher all-cause mortality risk compared to white-collar and professionals (HR=1.64, 95%CI: 1.19-2.27 and HR=1.55, 95%CI: 1.13-2.12, respectively).

The survival analysis of DNA methylation measures and clinical mediators and mortality are presented in Supplementary Table 2. Among the chronological age clocks, most of the clocks showed significant associations with all-cause mortality, and GrimAge, GrimAge2, and DunedinPoAm showed the strongest effect sizes with 1.11 (95%CI: 1.09-1.13), 1.10 (95%CI: 1.09-1.12), and 43.7 (95%CI:16.9-113), respectively. Weidner and SkinBlood epigenetic age showed less strong associations, and Yang epigenetic age and Horvath predicted telomere showed null effects. Only GrimAge, GrimAge2, and DunedinPoAm showed significant associations with both cardiovascular-specific and cancer-specific mortality due to smaller sample size. In addition, Hannum, Horvath, Vidal-Bralo, and PhenoAge epigenetic age showed significant associations with cancer-specific mortality with a HR range of 1.04 (95%CI: 1.01-1.07) to 1.05 (95%CI: 1.02-1.07).

### The mediating roles of DNA methylation measures and clinical factors in the association between race and ethnicity and all-cause mortality

The mediation analysis of DNA methylation clocks and clinical biomarkers for the association between race and ethnicity and all-cause mortality are shown in Figure 2 and Supplementary Table 3. Multiple mediation signals were observed when comparing Black and White participants, as well as for the comparison between Hispanic and White participants. Physiologically trained clocks GrimAge, GrimAge2, and DunedinPoAm showed moderate mediation. Among Black vs. White participants comparisons, GrimAge, GrimAge2, and DunedinPoAm mediated a proportion of 34% (95%CI: 19%-67%), 46% (95%CI: 27%-89%), and 24% (95%CI: 10%-49%), respectively. Unexpectedly, all chronological age clocks except Lin epigenetic age exhibited significant negative mediation ranging from -3% (95%CI: -8%- -0.3%) to -19% (95%CI: -38%- -10%), and Horvath predicted telomere exhibited -44% (95%CI: -79%- -27%) mediation. These results indicated that although compared to White participants, Black participants had overall higher risk of mortality, they also had on average higher Horvath predicted telomere values. These higher values of DNA methylation predicted telomere length are associated with reduced risks for mortality and protective for Black participants to show even greater mortality disparities. Comparing Hispanic to White participants, GrimAge2 and DunedinPoAm pace of aging exhibited significant positive mediation of 39% (95%CI: 18%-101%) and 28% (95%CI: 13%-70%), respectively. Most chronological age clocks did not exhibit significant mediation effects, except for Vidal-Bralo, which showed a significant negative mediation effect of -11% (95%CI: -44%- -2.3%). No DNA methylation or clinical biomarkers demonstrated significant mediation effects in the comparison between other races and White participants due to small sample size.

**Figure 2:**
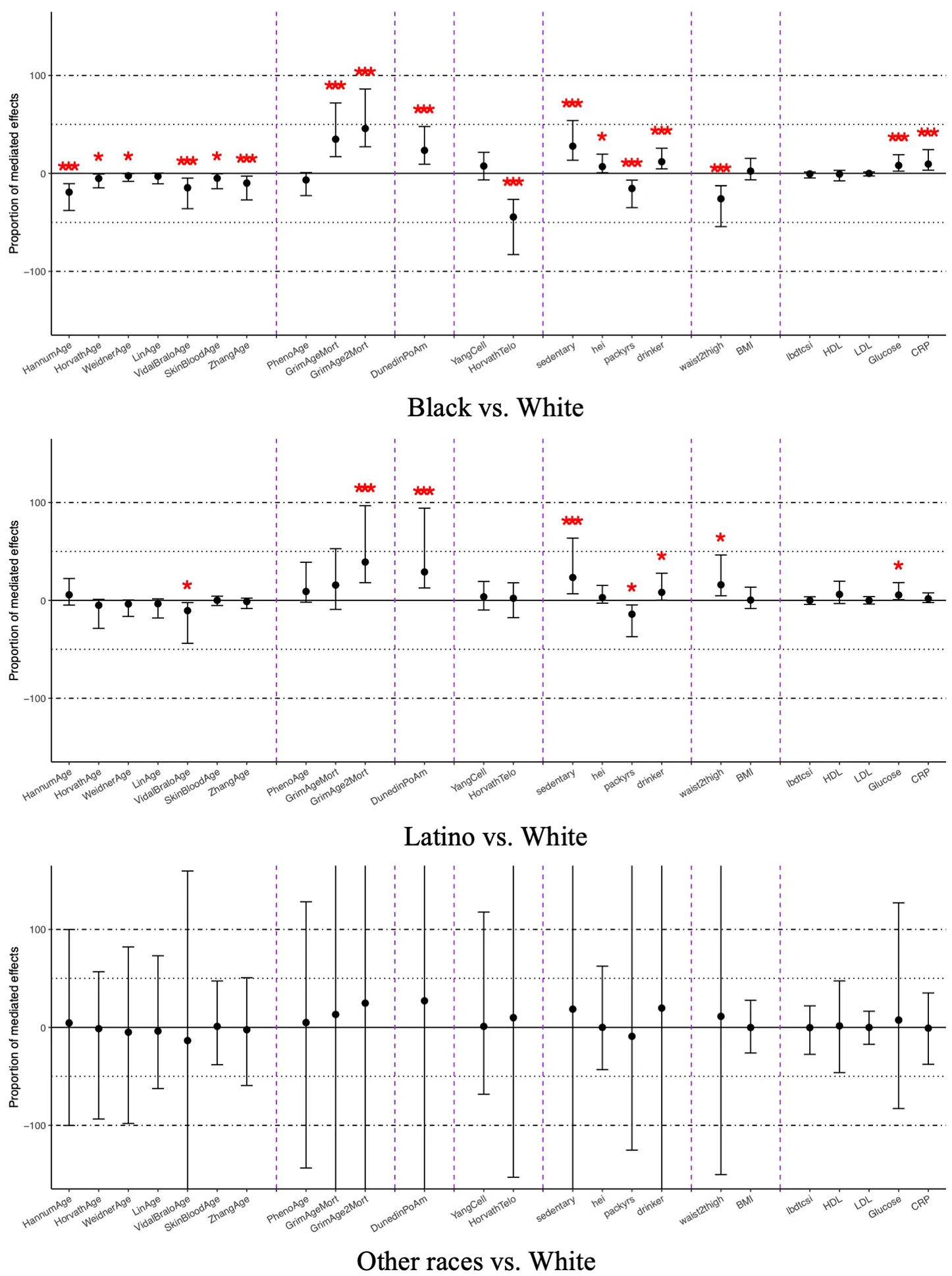
**Average mediated proportions for DNA methylation and clinical mediators on the relationship between race and ethnicity and all-cause mortality, NHANES 1999-2002 (n=2,402).** Coefficients marked with one asterisk indicate statistical significance (p < 0.05), and those with three asterisks indicate significance after Bonferroni correction (p < 0.0021). The x-axis is ordered as chronological age clocks, physiological functioning clocks, pace-of-aging clock, clocks trained on other aging biomarkers, and behavioral, anthropometric, and clinical mediators.

### The mediating roles of DNA methylation measures and clinical factors in the association between education and all-cause mortality

The mediation analysis for educational mortality disparities is shown in Figure 3 and Supplementary Table 4. The patterns of mediation were consistent across all comparisons of educational attainment levels. Physiologically trained clocks showed stronger effects compared to other types of clocks. GrimAge, GrimAge2, and DunedinPoAm epigenetic age exhibited significant mediation effects across all comparisons, ranging from 11% (95%CI: 2.8%-28%) to 32% (95%CI: 17%-64%), which were higher than the smoking pack-years, the leading clinical biomarkers. Horvath predicted telomere mediated 5.3% (95%CI: 1.7%-10%) and 5.7% (95%CI: 1.9%-13%) of the mortality disparities for participants with less than a high school education and those with a high school degree or educational development program (GED), respectively. In addition, PhenoAge epigenetic age mediated 6.5% (95%CI: 2.4%-14%) of the difference between participants with less than a high school education and those with a college degree, and Hannum epigenetic age showed a marginally significant positive mediation of 4.4% (95%CI: 0.3%-13.4%) between participants with a high school degree or GED and those with a college degree or higher.

**Figure 3:**
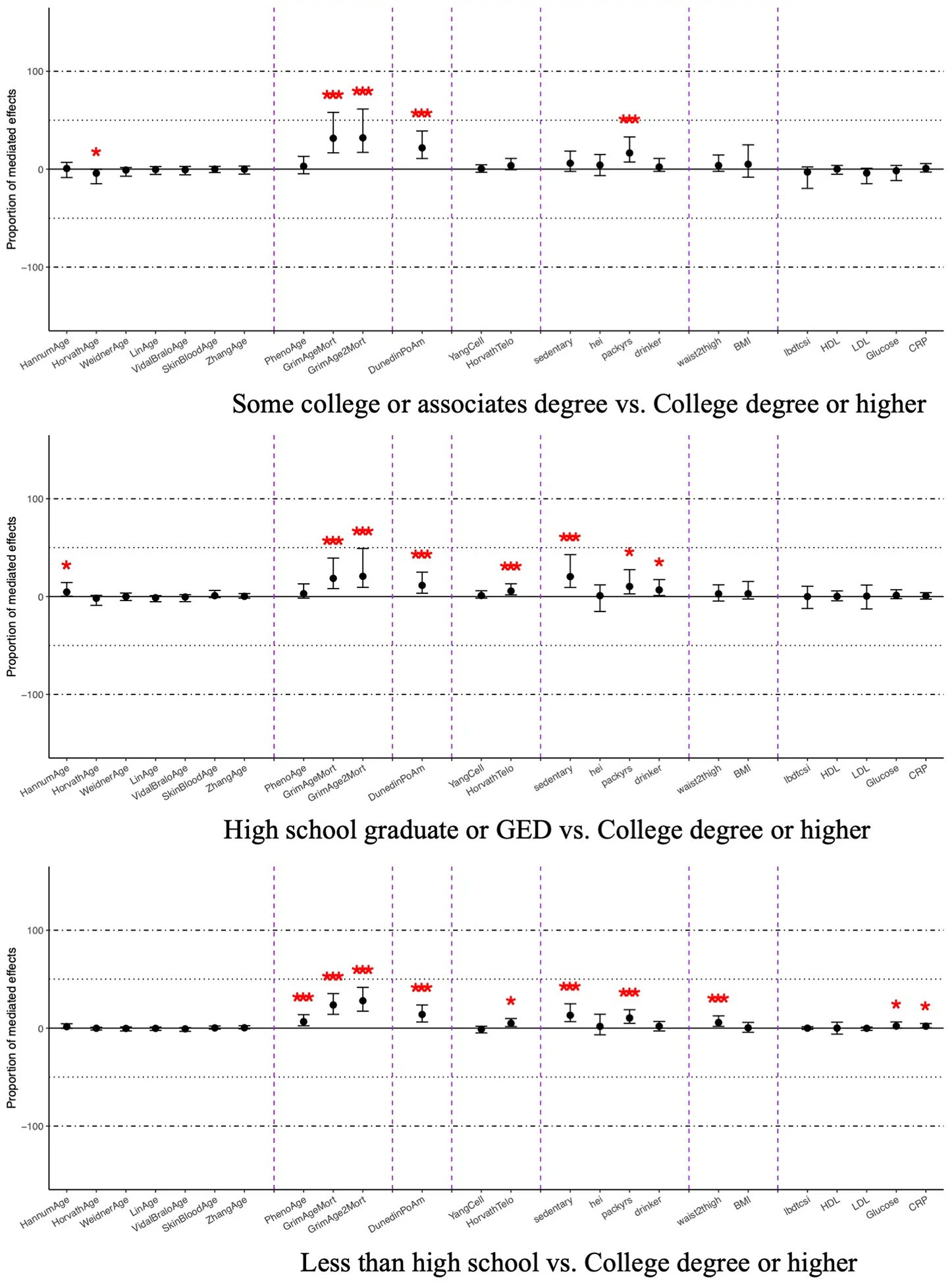
**Average mediated proportions for DNA methylation and clinical mediators on the relationship between education attainment and all-cause mortality, NHANES 1999-2002 (n=2,402).** Coefficients marked with one asterisk indicate statistical significance (p < 0.05), and those with three asterisks indicate significance after Bonferroni correction (p < 0.0021). The x-axis is ordered as chronological age clocks, physiological functioning clocks, pace-of-aging clock, clocks trained on other aging biomarkers, and behavioral, anthropometric, and clinical mediators.

### The mediating roles of DNA methylation measures and clinical factors in the association between household income and all-cause mortality

The mediation analysis for income-related disparities in all-cause mortality is presented in Figure 4 and Supplementary Table 5. The results followed patterns similar to those observed for educational attainment. PhenoAge, GrimAge, GrimAge2, Horvath predicted telomere, and DunedinPoAm showed significant positive proportion of mediation effects with smaller effects compared to the results from education attainment, but still greater effects compared to all the clinical mediators. GrimAge, GrimAge2, and DunedinPoAm epigenetic age significantly mediated effects across all group comparisons ranging from 14% (95%CI: 6.6%-29%) to 37% (95%CI: 22%-58%). In addition, PhenoAge and Horvath predicted telomere significantly mediated part of the group comparisons, ranging from 3.7% (95%CI: 0.3%-11%) to 8.3% (95%CI: 2.5%-17%).

**Figure 4:**
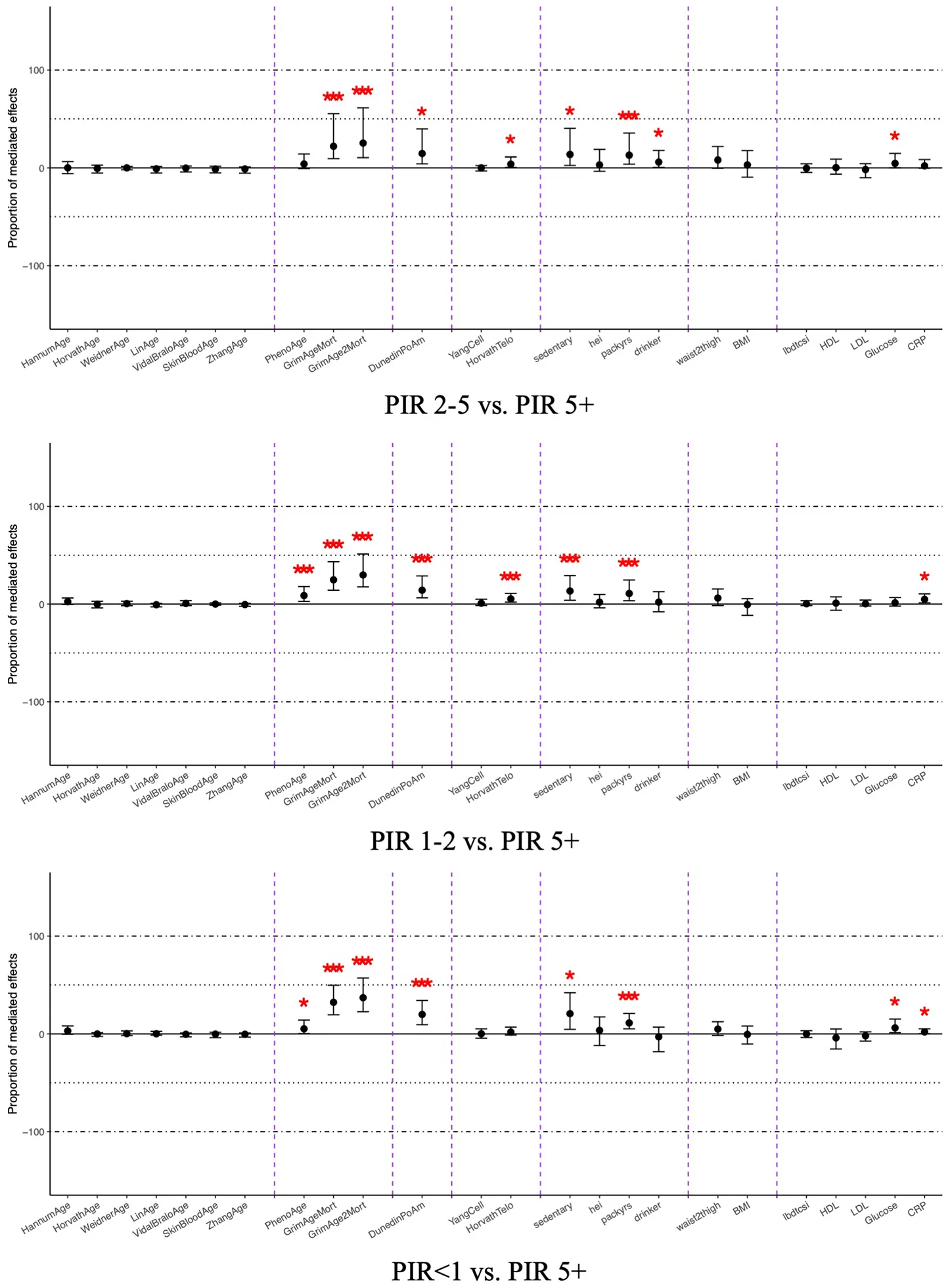
**Average mediated proportions for DNA methylation and clinical mediators on the relationship between household income and all-cause mortality, NHANES 1999-2002 (n=2,402).** Coefficients marked with one asterisk indicate statistical significance (p < 0.05), and those with three asterisks indicate significance after Bonferroni correction (p < 0.0021). The x-axis is ordered as chronological age clocks, physiological functioning clocks, pace-of-aging clock, clocks trained on other aging biomarkers, and behavioral, anthropometric, and clinical mediators.

### The mediating roles of DNA methylation measures and clinical factors in the association between occupation and all-cause mortality

The mediation of DNA methylation clocks on occupation-related disparities is shown in Figure 5 and Supplementary Table 6. Compared to the white-collar and professional participants, individuals in semi-routine white-collar and those who had never worked did not show significant mediations of any DNA methylation clocks and clinical biomarkers. For the comparison between high-skilled and semi-routine blue-collar and white-collar/professional participants, physiologically trained clocks again showed stronger effects compared to other types of clocks and clinical mediators. GrimAge, GrimAge2, and DunedinPoAm epigenetic age mediated 25% (95%CI: 14%-67%) up to 52% (95%CI: 26%-128%). In addition, Hannum, PhenoAge, and Horvath predicted telomere mediated 10% (95%CI: 2.2%-27%), 9.0% (95%CI: 2.1%-35%), and 6.1% (95%CI: 0.9%-16%) of the mortality disparities, respectively, when comparing high-skilled blue-collar workers to white-collar professionals.

**Figure 5:**
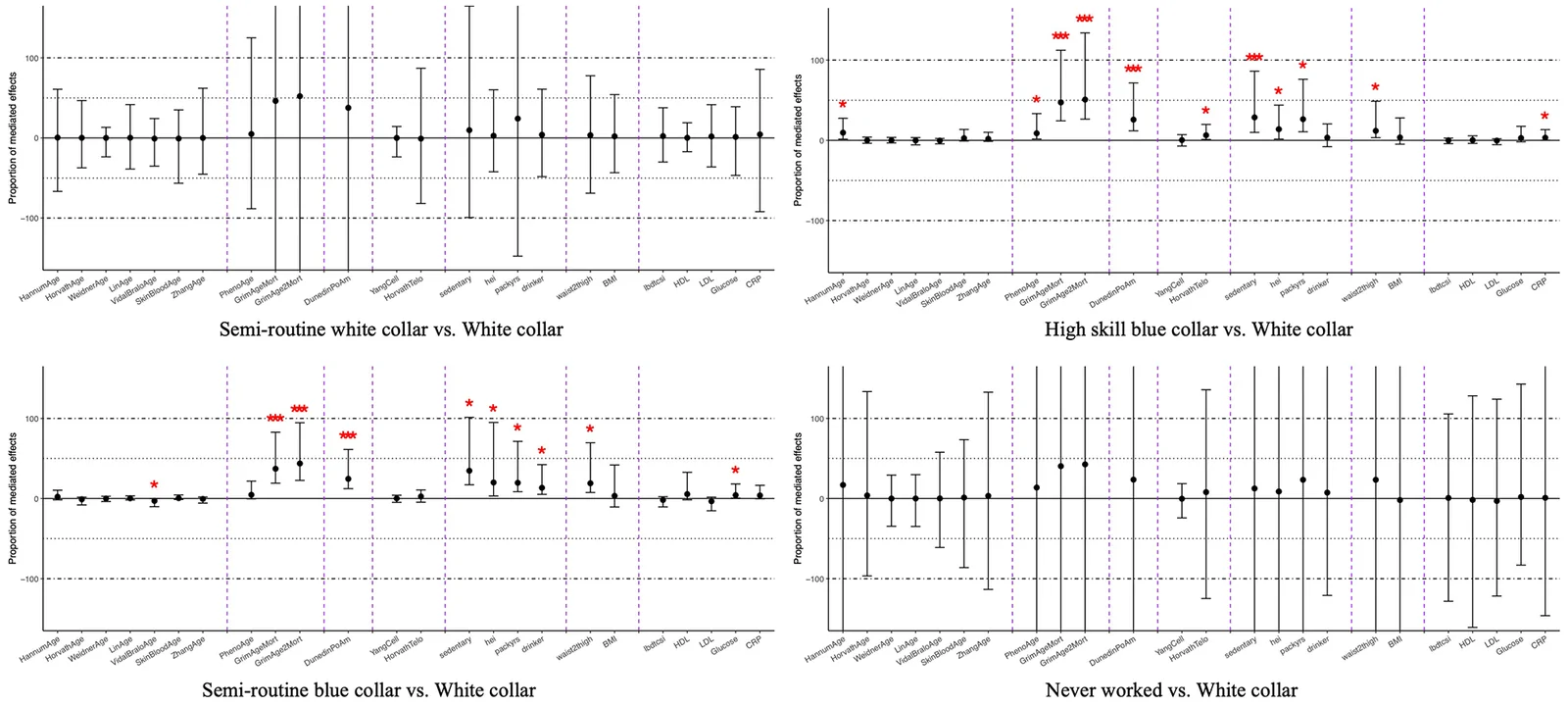
**Average mediated proportions for DNA methylation and clinical mediators on the relationship between occupation status and all-cause mortality, NHANES 1999-2002 (n=2,402).** Coefficients marked with one asterisk indicate statistical significance (p < 0.05), and those with three asterisks indicate significance after Bonferroni correction (p < 0.0021). The x-axis is ordered as chronological age clocks, physiological functioning clocks, pace-of-aging clock, clocks trained on other aging biomarkers, and behavioral, anthropometric, and clinical mediators.

### Secondary analysis of cause-specific mortality disparities

The exposure survival analysis for cause-specific mortality is also presented in Supplementary Table 1. For cardiovascular-specific mortality, due to the smaller simple size, only the comparisons between Black and White participants and between participants with a poverty income ratio (PIR) of less than 1 and those with a PIR of 5 or above remained significant with a HR of 2.12 (95%CI: 1.25-3.58) and 4.33 (95%CI: 1.63-11.51), respectively. For cancer-specific mortality, only models for educational attainment and occupational status showed significant results. Compared to participants with a college degree or higher, those with less than a high school education (HR=3.53, 95%CI: 1.43-8.70), a high school diploma or GED (HR=3.21, 95%CI: 1.41-7.29), and some college (HR=3.34, 95%CI: 1.52-7.33) had significantly higher risks. Similarly, participants in high-skilled blue-collar (HR=2.63, 95%CI: 1.50-4.63) and semi-routine blue-collar (HR=1.95, 95%CI: 1.13-3.34) occupations had significantly higher cancer-specific mortality risks compared to white-collar professionals. The mediator survival analysis for both cardiovascular- and cancer- specific mortality are shown in Supplementary Table 2. GrimAge, GrimAge2, and DunedinPoAm epigenetic age exhibited significant effects similar to the all-cause mortality results. For cancer-specific mortality only, more clocks showed significant associations, with HRs ranging from 1.04 (95%CI: 1.01-1.07) to 1.05 (95%CI: 1.02-1.07), including Hannum, Horvath, Vidal-Bralo, and PhenoAge.

The mediation analysis for cause-specific mortality had lower statistical power than for all-cause mortality (Supplementary Tables 7-14). For racial disparities, comparing Black and White participants, GrimAge, GrimAge2, and DunedinPoAm epigenetic age significantly mediated 17% (95%CI: 6.6%-56%), 23% (95%CI: 8.7%-63%), and 10% (95%CI: 2.0%-37%) of the cardiovascular-specific mortality disparity, respectively, though these effects were smaller than that of sedentary behavior (mediated proportion=26%; 95%CI: 11%-92%) (Supplementary Table 7). However, for cardiovascular disparities between those with a PIR <1 and those with a PIR ≥5, GrimAge and GrimAge2 epigenetic age mediated 23% (95%CI: 11%-50%) and 26% (95%CI: 11%-62%) which were higher than other clinical mediators (Supplementary Table 9). In cancer-specific mortality disparities, GrimAge, GrimAge2, and DunedinPoAm epigenetic age also demonstrated significant mediation for education- and occupation-related disparities, with mediated proportion ranging from 6.5% (95%CI: -3.4%-21%) to 30% (95%CI: 12%-66%). These three DNA methylation epigenetic age measures mediated even larger proportions for income-related cancer-specific mortality disparities with 76% (95%CI: 30%-326%), 80% (95%CI: 28%-416%), and 54% (95%CI: 12%-241%), respectively (Supplementary Table 13).

### Sensitivity analysis of all-cause mortality stratified by subgroups

The mediation analysis stratified by gender is shown in Supplementary Tables 15–22. Although no significant difference was found, it is interesting to notice that the mediation signals for all-cause mortality disparities between Hispanic and White participants and people from different levels of household incomes were primarily driven by women, in comparison to all-cause mortality disparities between Black and White participants, people from different levels of education attainments, and individuals from different levels of occupation status were driven by men. The mediation analysis stratified by race and ethnicity is presented in Supplementary Tables 23–31. Again, although no significant difference shown across different races and ethnicities, DNA methylation biomarkers showed the most signals among White participants and then followed by Black participants. No significant signals were observed for Hispanic participants.

For more sensitivity analysis, we provided the results for all ten components of GrimAge2 in Supplementary Tables 32–35. These results showed that overall mediation effects of GrimAge2 were larger than that of any individual component, with the pack-years component showing the strongest effect among them. We also provided sensitivity analysis using completed cases only in Supplementary Tables 36–39. The results showed similar patterns compared to our main results using the imputed dataset. In addition, we provided testing results for exposure-mediator interaction and non-linear square term effects of each mediator on all-cause mortality in Supplementary Tables 40, 41. As the results demonstrated there was no consistent evidence across groups showing significant interactions between exposures and mediators in Supplementary Table 40. Supplementary Table 41 also suggested there was no evidence showing any non-linear effects of DNA methylation clocks on the all-cause mortality outcomes, although among clinical mediators, BMI, total cholesterol, and C-reactive protein presented significant square terms with betas that ranged from -0.037 to 0.066.

## DISCUSSION

Aiming to systematically examine the mediating role of DNA methylation clocks in the associations between race, ethnicity, education, income, and occupation and mortality**,** this study uses nationally representative data to demonstrate that DNA methylation clocks, particularly GrimAge2 and DunedinPoAm, mediate a substantial proportion of racial/ethnic and socioeconomic disparities in mortality, consistent with our hypothesis. GrimAge2 exhibited significant mediation on all-cause mortality disparities, accounting for 21% of the difference between participants with a high school diploma or GED and those with a college degree or higher, up to 52% of the difference between individuals in high-skilled blue-collar occupations and those in white-collar and professional positions. Similarly, the DunedinPoAm pace of aging mediated 11% of the mortality disparity between high school graduates and individuals with a college degree or above, and 28% of the disparity between Hispanic and White participants. Notably, these mediation results, particularly for GrimAge2, were greater than those observed for traditional clinical biomarkers. These findings suggest that DNA methylation clocks and biomarkers could serve as valuable tools for future research investigating the mechanisms underlying health disparities and for the more rapid evaluation of the potential long-term impacts of policy changes.

Results supporting our hypothesis that DNA methylation clocks are good biomarkers for mortality outcomes, especially GrimAge2, are consistent with previous literatures. A few studies have explored the mediating effects of GrimAge and GrimAge2 on mortality disparities. A 2022 meta-analysis integrating data from 18 cohorts across multiple countries found that GrimAge mediated 60% of the mortality disparity between men with lower than upper secondary education and those with upper secondary education or higher, an effect stronger than that observed for other clocks, including Hannum, Horvath, and PhenoAge [24]. Similarly, a study using data from Framingham Heart Study reported that GrimAge mediated 63% of the association between education attainment and all-cause mortality, again exceeding the mediation effects observed for other clocks [27]. Although these studies have suggested that much of the mediation effects could be attributed to differences in unhealthy lifestyles, such as smoking, alcohol consumption, high BMI, low physical activity, and chronic conditions including hypertension and diabetes, our study extends this literature by directly comparing the mediation effects between GrimAge and GrimAge2 with those of traditional clinical biomarkers within the same dataset. In addition, we demonstrate that GrimAge2 reported greater mediated proportions for social inequalities in mortality disparities than any single clinical measure. For cause-specific mortality, one study found that GrimAge mediated up to 24% of the association between household income and cardiovascular mortality, a larger effect than that observed for other causes of death [26]. In contrast, our analyses show that both GrimAge and GrimAge2 mediated a significant portion of all-cause, cardiovascular-specific, and cancer-specific mortality with the largest proportion observed for cancer mortality disparities between people living under poverty and people with PIR ≥5, up to 80%. This difference may reflect variation in study populations, as the prior study was restricted to participants with cardiovascular-kidney-metabolic syndrome, suggesting that the GrimAge clock may perform more strongly among healthier populations.

Regarding the performance of other DNA methylation clocks, Graf et al. [27] found that DunedinPACE (a later version of DunedinPoAm) mediated 50% of the association between education attainment and all-cause mortality. In comparison, our study found DunedinPoAm mediated 11% to 22% of this association. This difference may reflect the use of an earlier version of the Dunedin clock in our analysis. In addition, Fiorito and colleagues [24] reported that the Horvath, Hannum, and PhenoAge epigenetic age mediated 21%, 26%, and 33% of education-related mortality disparities among men, respectively. Similarity, a study using data from Women's Health Initiative found that PhenoAge mediated 12% of mortality disparities by race (Black vs. White) and 16% by educational attainment (less than high school vs. college degree or higher) among women [25]. In our study, PhenoAge significantly mediated 6.6% of the mortality disparity associated with lower educational attainment (less than high school vs. college degree or higher) but showed no significant mediation ability for other social factors. Together, these findings may suggest that some epigenetic clocks, especially the clocks trained on chorological age, may have limited mediation utility when generalized to nationally representative populations.

Our study has implications for why DNA methylation biomarkers showed a wide range of mediated effects on social inequalities, which is not consistent with our original hypothesis. As demonstrated by the correlation analysis in our study, DNA methylation biomarkers carry different aspects of signals rather than belonging to one cluster of epigenetic information. Although physiologically trained clocks and the pace of aging clock performed better in the mediation analysis (particularly GrimAge2 and DunedinPoAm), clocks trained on chronological age, cancer biomarkers, or telomere length may still provide complementary information worthy of future investigation. Interestingly in our results, some of these clocks negatively mediated the all-cause mortality disparities between Black and White participants, especially Horvath predicted telomere mediated -44%. One explanation could be the protective role of telomere-related mechanisms in racial morality disparities. While no prior studies have directly examined telomere length as a mediator of racial health disparities in mortality, existing evidence supports this hypothesis. As well documented in previous literature, longer leukocyte telomere length is associated with lower risk of mortality [28–31]. A number of studies also have reported that Black adults, on average, have longer telomeres than their White counterparts [32, 33]. Building on this evidence, our findings imply that for Black and White Americans with equivalent telomere lengths, racial disparities in mortality might be even greater than those observed in studies without accounting for telomere length. Another explanation could be that these biomarkers are associated with other important variables on the pathway. For example, both Hannum epigenetic age and Horvath predicted telomere were suggested on the causal pathway of social support affecting mortality risks [34]. African American may have on average stronger social connections, which is protective for them to counter racial disparities in mortality. These hypotheses suggest that African Americans with shorter telomeres and low social connections may represent a particularly vulnerable subgroup for mortality disparities.

In addition, consistent with our hypothesis, our results indicate that DNA methylation can potentially be developed as a better composite biomarker for measuring biological embeddedness for social disadvantages in the future. Previous studies have explored the effects of clinical variables in cohorts from various countries showing clinically measured variables, including diagnoses of chronic diseases, even combing can only mediate up to moderate effects of mortality disparities originated from social stratifications [35–38]. For example, a previous study combining 179,090 participants reported a joint mediation of smoking, alcohol consumption, dietary patterns, physical activity, BMI, hypertension, diabetes, and coronary artery disease, account 34% and 38% in women and men separately [35]. Therefore, our results showing DNA methylation biomarkers mediated a bigger proportion than these effects suggest promising utility for capturing social health inequalities.

This study examined the mediation effects of thirteen DNA methylation epigenetic biomarkers on mortality disparities across multiple individual social stratification indicators, including race, ethnicity, educational attainment, household income, and occupation, by taking advantage of a nationally representative probability-based sample. However, several limitations should be noted. First, although causal mediation models were used, the cross-sectional design of this study limits causal inference. While socioeconomic factors likely preceded DNA methylation measures, our results remain vulnerable to potential reverse causality of the effects flowing from health to socioeconomic position, particularly income [8]. For example, worse health may lead to reduced labor force participation and, in turn, lower income [39]. Due to the cross-sectional design of our study, we could not differentiate directionality in our findings. Further studies with longitudinal design are needed to validate our results related to household income. Second, studies have suggested that factors such as gender, marital status, and birth year may modify the effects of mortality disparities associated with socioeconomic positions [40]. Previous research also has shown DNA methylation clocks can perform differently by gender and race [24, 41]. Although our results did not show any significant modification effects by gender and race, it still needs to be cautiously interpreted among different subgroups. Further studies with larger sample sizes should explore subgroup differences and the underlying mechanisms of these modification effects. Third, as our study sample was restricted to individuals aged 50 to 84, the findings may not be generalized to younger population. Additional research is needed to investigate whether similar DNA methylation-based mediation effects can be observed in younger cohorts. Fourth, this study only conducted single mediation analysis for each individual epigenetic and clinical biomarker due to the limits of our current methods used. Future studies are needed to explore combining the mediation ability across these epigenetic, clinical biomarkers and other potential candidate mediators with advanced statistical methods to better capture the complexity of the associations. Fifth, recent literature has indicated using packages of multiple imputation and mediation with confidence intervals calculated by non-parametric bootstrap method at the same time may introduce potential biases [42, 43]. The exact values of our estimates may need to be interpreted with caution even though the main analysis and complete case analysis showed same patterns. Sixth, our study has only considered linear terms for the mediators due to the limits of our modelling method. Studies have suggested some mediators, such as BMI [44], can have potential nonlinear effects on mortality outcomes. Future studies should use complex mediation models to explore the nonlinear effects for these biomarkers. Seventh, our study compared the effects on a relative scale but not on the absolute scale. Absolute inequalities are important for public health interventions because it can provide further information on the scale of the impacts on population. Future studies should explore if the estimates would be different by absolute scale. Lastly, genetic ancestries may serve as an important confounder influencing both exposures and DNA methylation biomarkers, given their strong correlation with self-reported race and ethnicity. Because genetic ancestry information was not available in this dataset, it remains uncertain how this unmeasured factor may have shaped our findings. Future research incorporating genetic ancestry data could help clarify the extent to which observed associations reflect underlying biological variation versus socially patterned differences that covary with ancestry and race.

In conclusion, this study contributes to the growing body of evidence demonstrating that DNA methylation clocks mediate a substantial proportion of mortality disparities associated with adverse social exposures, with stronger effects than traditional clinical biomarkers. These findings also underscore the potential of DNA methylation measures as valuable tools for understanding the biological embedding of social disadvantages. Further research using longitudinal study designs and larger sample sizes is needed to validate these results and clarify the mechanisms underlying these associations. As more insights emerge regarding the role of DNA methylation clocks and biomarkers, these measures may prove useful in future studies evaluating the broader impact of social policies and identifying vulnerable populations for targeted long-term disease prevention efforts.

## MATERIALS AND METHODS

### The NHANES participants

We analyzed the participants from the U.S. National Health and Nutrition Examination Survey (NHANES) 1999-2000 and 2001-2002 cycles who had publicly available DNA methylation clock measurements. Eligible participants were non-Hispanic White, non-Hispanic Black, Hispanic, and other races (including multi-racial participants), aged 50 years or older who had a blood sample collected at baseline and consented to future genetic research. Further details on the methodology of DNA methylation data are available on the NHANES website [45]. Participants were included in this study if they had non-zero DNA methylation specific weights (N=2,532). We excluded individuals who were 85 years and older (N=130) since NHANES does not report the exact ages for this group. After exclusion, the final study sample consisted of 2,402 participants.

### Socioeconomic position measures

Socioeconomic position measures were derived from NHANES baseline questionnaires, with all variables being self-reported and publicly available. Educational attainment was assessed as the highest self-reported level of education completed by participants. This variable was classified as less than high school, high school graduate or general educational development program (GED), some college or an associate’s degree, and a college degree or higher.

Household income was measured using the poverty income ratio (PIR), defined as the ratio of a participant’s household income to the federal poverty levels from Department of Health and Human Services’ (HHS) poverty guidelines. To facilitate comparisons, household income was categorized into four groups: below the poverty line (PIR < 1), PIR 1-2, PIR 2-5, and PIR ≥ 5 for comparison with other work [46, 47].

The longest held occupation of the participants was used to define their occupational status. When data on the longest held occupation was missing, the reported current occupation was used to fill the missing values. Occupational status was categorized into five groups: white-collar and professional, semi-routine white-collar, high-skilled blue-collar, semi-routine blue-collar, and never worked, according to the National Statistics Socio-Economic Classification [48].

### Epigenetic aging and clinical mediators

Thirteen DNA methylation clocks available in NHANES were included in this analysis. Clocks trained on chronological age included Hannum [49], Horvath [50], Weidner [51], Lin [52], VidalBralo [53], SkinBlood [54], and Zhang [55]. Clocks trained to predict morbidity or mortality included PhenoAge [56], GrimAge [57], and GrimAge2 [58]. In addition, the pace of aging clock DunedinPoAm [59], which was trained on aging rates measured across multiple organ systems, was included. Other clocks trained on aging biomarkers such as Yang [60] which was trained on cancer biomarkers, and Telomere [61] which was trained on leukocyte telomere length were also included.

To assess the mediation effects for traditional clinical measures from established biological pathways, additional clinical measures were included in tests of proportion mediated. For behavioral variables, we included physical activity (sedentary vs. active), the 2015 health eating index (HEI) [62], smoking pack-years, and drinking behaviors (abstainer vs. alcohol drinker). Anthropometric measures included waist-to-thigh ratio and body mass index (BMI). Clinical biomarkers included total cholesterol, high-density lipoprotein (HDL), low-density lipoprotein (LDL), glucose, and C-reactive protein (CRP). Physical activity status was defined as sedentary if participants did not report any vigorous, moderate, or muscle-strengthening activities over the past 30 days [63].

### Mortality outcomes and covariates

Mortality data were obtained from the National Center for Health Statistics publicly available mortality files, which track deaths through linkage with the National Death Index (NDI). Participants were classified as deceased if their records were linked to the NDI by December 31, 2019, and were assumed to be alive otherwise. The underlying cause of death as recorded in the NDI has been validated with an estimated classification error of approximately 5% [64]. The maximum follow-up period was 17 years. The age at death or loss of follow-up was used as the survival outcome. All-cause mortality was examined as the primary event with loss to follow-up considered as censored, while cause-specific mortality from either cardiovascular disease or cancer were evaluated as the secondary and exploratory event with all other deaths and loss to follow-up both considered as censored. As shown in the directed acyclic graph (DAG) presented in Supplementary Figure 1, covariates controlled in the model included gender (women vs. men), baseline age and baseline age^2^ (to account for potential non-linear effects of age on predictor and outcome variables), and nativity (U.S.-born vs. foreign-born).

### Statistical modeling

Descriptive analyses were performed using means and standard deviations for continuous variables, and frequencies with percentages for categorical variables. A nonparametric random forest multiple imputation method was used to impute the missingness in exposures, covariates, and clinical mediators (*missForest* package in R [65, 66]). All the participants from 1999-2002 cycle (N = 21,004) were used to impute missing values. This package is designed to handle data that are Missing at Random (MAR). Correlation analyses to show the clusters across all DNA methylation biomarkers were conducted using R functions *rcorr*. Survival analyses were conducted using a Weibull model implemented in the *survey* package in R, incorporating NHANES DNA methylation specific survey weights to account for the complex survey design. Hazard ratios (HR) and 95% confidence intervals (CI) were reported. Only linear terms of mediators were included in the models, and Wald-test was used to test the non-linear square term effects of mediators in the associations between mediator and outcome.

Mediation analyses were conducted using the *mediation* package in R [67], estimating average mediated proportions along with their 95% CIs. This package outputs mediation proportions ranging from negative infinity to positive infinity. A negative mediated proportion indicates that the exposure and mediator have effects on the outcome in opposite directions, whereas a mediated proportion greater than 100% indicates that the mediator captures an effect in the same direction that is larger than the total effect of the exposure on the outcome. Exposure may influence an outcome through multiple pathways, producing both beneficial and detrimental effects that operate in different directions. As a result, the overall effect estimate may be attenuated relative to the true causal effect in that direction. This phenomenon is particularly relevant for social exposures, as some adverse social conditions may also be associated with social cohesion and resilience [5, 14]. A more detailed discussion is included in Appendix 1.

This mediation model uses a potential outcome framework to estimate mediation effects. We used a parametric approach within this package, which means the estimates will be calculated from the regression parameters and the standard errors will be calculated from bootstrap method. The assumptions of this approach include 1) no unobserved pre-treatment confounding between social positions and mortality outcomes besides the covariates already controlled; 2) no unobserved pre-treatment and post-treatment confounding for the associations of DNA methylation clocks and mortality outcomes; 3) sufficient overlap in the distributions of social position and DNA methylation clocks across covariates. A more detailed discussion of how well our study met the assumption is included in Appendix 2. The package built-in function *test.TMint* was used to test if there were interactions between exposures and mediators. Results with a p-value of <0.05 were considered as marginally significant, and a p-value of <0.0021 (0.05/24, as there was total 24 mediators included in the study) were considered as significant results that passed multiple testing. Sensitivity analyses conducted among different gender and racial subgroups were also presented. All statistical analyses were performed using R version 4.5.0. The R code and data used for these analyses are provided in a GitHub repository (https://github.com/shywatson/SES_DNAm_NHANES).

## Supplementary Material

Supplementary Material

Supplementary Figure 1

Supplementary Table 1

Supplementary Tables 2-5

Supplementary Table 6

Supplementary Tables 7-9

Supplementary Table 10

Supplementary Tables 11-13

Supplementary Table 14

Supplementary Tables 15-20

Supplementary Table 21

Supplementary Table 22

Supplementary Tables 23-28

Supplementary Table 29

Supplementary Table 30

Supplementary Table 31

Supplementary Tables 32-34

Supplementary Table 35

Supplementary Tables 36-38

Supplementary Table 39

Supplementary Table 40

Supplementary Table 41
