# Supplementary material for: The mediating role of DNA methylation clocks in associations of race, ethnicity, education, income, and occupation with mortality: findings from NHANES 1999-2002
Source: Aging (Albany NY). 2026 May 8;18(1):466–512. doi: 10.18632/aging.206377 (PMC13632862; doi:10.18632/aging.206377)
Supplement: Supplementary file 1 [file aging-18-1-206377-s002.pdf]

SUPPLEMENTARY FIGURE

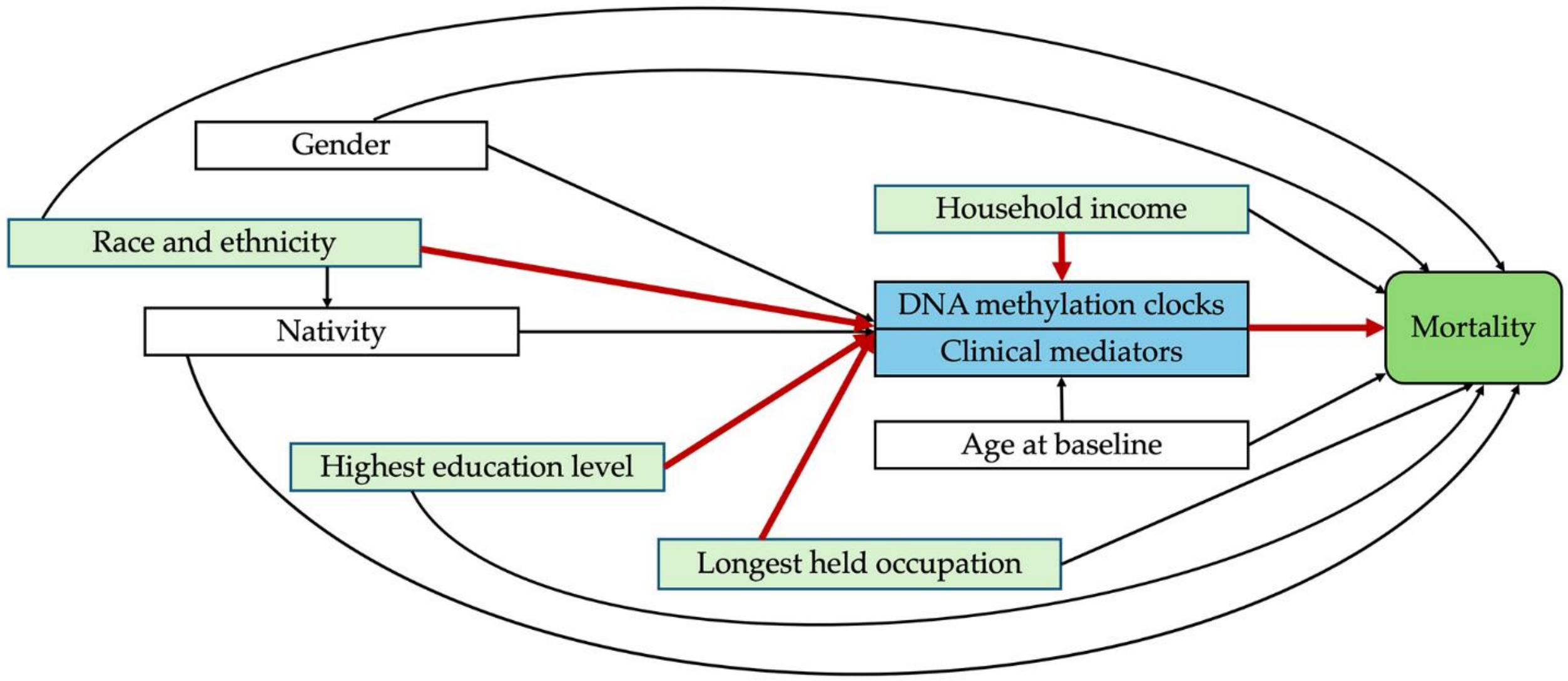

Supplementary Figure 1. Directed acyclic graph (DAG) illustrating the variables included in the study. Red arrows indicate the primary pathways examined in the analysis. Light green boxes represent the main exposures, and the dark green box represents the main outcome. Blue boxes denote mediators, while white boxes represent covariates adjusted for in the models.
