# Supplementary material for: The mediating role of DNA methylation clocks in associations of race, ethnicity, education, income, and occupation with mortality: findings from NHANES 1999-2002
Source: Aging (Albany NY). 2026 May 8;18(1):466–512. doi: 10.18632/aging.206377 (PMC13632862; doi:10.18632/aging.206377)
Supplement: Supplementary file 2 [file aging-18-1-206377-s004.pdf]

Supplementary Table 2. Survival analysis for DNA methylation clocks and clinical biomarkers on all-cause mortality, controlled by gender, age, age2, and nativity, NHANES 1999-2002 (n=2,402). Bold indicates statistical significance (p-value<0.05).

	All-cause mortality			Cardiovascular-specific mortality			Cancer-specific mortality		
	HR	(95% CI)		HR	(95% CI)		HR	(95% CI)	
**Chronological age clocks**									
Hannum	**1.04**	**(1.02**	**1.05)**	1.01	(0.97	1.05)	**1.05**	**(1.02**	**1.07)**
Hovarth	**1.03**	**(1.01**	**1.04)**	1.00	(0.98	1.03)	**1.04**	**(1.02**	**1.07)**
Weidner	1.01	(1.00	1.02)	1.00	(0.98	1.02)	1.01	(0.99	1.03)
Lin	**1.01**	**(1.00**	**1.02)**	1.00	(0.98	1.02)	1.01	(0.99	1.03)
Vidal-Bralo	**1.02**	**(1.01**	**1.04)**	1.00	(0.97	1.04)	**1.04**	**(1.02**	**1.07)**
SkinBlood	1.02	(1.00	1.04)	1.01	(0.97	1.06)	1.01	(0.98	1.04)
Zhang	**1.06**	**(1.01**	**1.11)**	1.03	(0.93	1.14)	1.02	(0.95	1.09)
**Physiological functioning clocks**									
PhenoAge	**1.04**	**(1.03**	**1.05)**	0.99	(0.96	1.02)	**1.04**	**(1.01**	**1.07)**
GrimAge	**1.11**	**(1.09**	**1.13)**	**1.10**	**(1.06**	**1.14)**	**1.12**	**(1.07**	**1.16)**
GrimAge2	**1.10**	**(1.09**	**1.12)**	**1.09**	**(1.05**	**1.13)**	**1.10**	**(1.06**	**1.14)**
**Pace of aging clock**									
DunedinPoAm	**43.7**	**(16.9**	**113)**	**17.9**	**(2.37**	**136)**	**30.1**	**(5.52**	**164)**
**Clocks trained on other aging biomarkers**									
Yang	21.6	(0.23	2.03E+03)	120	(0.00	2.61E+08)	1.44	(0.00	1.14E+04)
Telomere	0.39	(0.29	0.51)	0.69	(0.28	1.67)	0.34	(0.18	0.66)
**Behavioral/Lifestyle measures**									
Sedentary behavior	**1.71**	**(1.45**	**2.01)**	**2.43**	**(1.67**	**3.52)**	1.31	(0.85	2.03)
Healthy eating index	**0.99**	**(0.98**	**1.00)**	1.00	(0.99	1.02)	**0.97**	**(0.96**	**0.99)**
Smoking pack years	**1.01**	**(1.01**	**1.01)**	**1.01**	**(1.00**	**1.01)**	**1.01**	**(1.01**	**1.02)**
Alcohol drinker	**0.75**	**(0.63**	**0.88)**	0.60	(0.38	0.94)	1.19	(0.78	1.83)
**Anthropometric measures**									
Waist to thigh ratio	**3.54**	**(2.18**	**5.76)**	**4.73**	**(1.26**	**17.8)**	1.79	(0.60	5.37)
BMI	1.01	(0.99	1.03)	1.00	(0.95	1.07)	1.00	(0.98	1.03)
**Clinical used blood Biomarkers**									
Total cholesterol	1.01	(0.94	1.08)	0.97	(0.75	1.25)	1.12	(0.93	1.34)
HDL	1.00	(0.99	1.00)	1.00	(0.99	1.02)	1.00	(0.99	1.01)
LDL	1.00	(1.00	1.00)	1.00	(0.99	1.00)	1.00	(1.00	1.01)
Glucose	**1.00**	**(1.00**	**1.01)**	**1.01**	**(1.00**	**1.01)**	1.00	(0.99	1.00)
C-reactive protein	**1.12**	**(1.06**	**1.17)**	1.04	(0.93	1.17)	**1.09**	**(1.01**	**1.18)**

Supplementary Table 3. Average mediated proportions for DNA methylation and clinical mediators on the relationship between race and ethnicity and all-cause mortality, NHANES 1999-2002 (n=2,402). Bold indicates statistical significance (p-value<0.05), and bold with two asterisks indicates significance passed Bonferroni adjustment (p-value<0.0021).

	Model 1. Black (N= 516) vs. White (N= 943)		Model 2. Hispanic (N=860) vs. White (N= 943)		Model 3. Other races (N=83) vs. White (N= 943)	
	Average mediated proportion	(95% CI)	Average mediated proportion	(95% CI)	Average mediated proportion	(95% CI)
**Chronological age clocks**						
Hannum	**-19.1%****	**(-37.8%- 10.5%)**	5.7%	(-4.9% 22.4%)	4.5%	(-100.1% 99.9%)
Hovarth	**-5.2%**	**(-14.6%- 0.7%)**	-5.0%	(-28.6% 1.1%)	-1.3%	(-93.6% 56.7%)
Weidner	**-2.5%**	**(-8.2%- 0.3%)**	-3.7%	(-16.4% 0.5%)	-4.9%	(-98.1% 82.1%)
Lin	-2.9%	(-10.6% 0.3%)	-3.4%	(-18.0% 1.6%)	-3.8%	(-62.5% 73.0%)
Vidal-Bralo	**-14.6%****	**(-35.9%- 4.9%)**	**-10.5%**	**(-43.9%- 2.3%)**	-13.5%	(-251.5% 159.5%)
SkinBlood	**-4.9%**	**(-15.5%- 0.5%)**	0.1%	(-5.2% 4.3%)	1.0%	(-38.1% 47.3%)
Zhang	**-9.9%**	**(-27.0%- 2.8%)**	-1.2%	(-8.3% 2.3%)	-2.4%	(-59.4% 50.6%)
**Physiological functioning clocks**						
PhenoAge	-6.8%	(-22.8% 0.6%)	8.9%	(-1.9% 37.8%)	5.7%	(-119.3% 161.6%)
GrimAge	**34.4%****	**(18.7% 66.8%)**	15.3%	(-9.8% 53.8%)	12.0%	(-193.5% 234.7%)
GrimAge2	**46.0%****	**(26.6% 88.7%)**	**39.3%****	**(18.2% 100.9%)**	24.8%	(-206.7% 288.7%)
**Pace of aging clock**						
DunedinPoAm	**23.6%****	**(10.0% 48.9%)**	**28.3%****	**(12.6% 70.4%)**	27.2%	(-211.4% 340.7%)
**Clocks trained on other aging biomarkers**						
Yang	7.4%	(-6.8% 20.9%)	3.6%	(-10.9% 21.1%)	1.3%	(-98.4% 124.6%)
Telomere	**-44.4%****	**(-79.1%- 27.2%)**	2.8%	(-21.6% 20.4%)	9.1%	(-177.4% 157.2%)
**Behavioral/Lifestyle measures**						
Sedentary behavior	**27.8%****	**(13.4% 54.0%)**	**23.4%****	**(6.7% 63.6%)**	18.5%	(-217.7% 298.3%)
Healthy eating index	**6.9%**	**(0.6% 19.6%)**	2.9%	(-2.8% 15.3%)	0.0%	(-43.2% 62.5%)
Smoking pack years	**-15.4%****	**(-34.8%- 6.9%)**	**-14.1%**	**(-37.1%- 4.6%)**	-9.1%	(-125.4% 170.8%)
Alcohol drinker	**11.9%**	**(4.6% 25.7%)**	**8.2%**	**(0.3% 27.8%)**	19.6%	(-309.1% 358.3%)
**Anthropometric measures**						
Waist to thigh ratio	**-25.8%**	**(-54.2%- 12.6%)**	**15.9%**	**(4.7% 46.3%)**	11.1%	(-150.4% 251.1%)
BMI	2.3%	(-6.6% 15.3%)	0.3%	(-8.3% 13.5%)	-0.1%	(-26.1% 27.6%)
**Clinical used blood Biomarkers**						
Total cholesterol	-0.5%	(-4.7% 1.4%)	0.0%	(-4.1% 3.7%)	-0.2%	(-27.5% 21.8%)
HDL	-0.8%	(-7.7% 3.1%)	6.2%	(-3.2% 19.6%)	1.6%	(-46.2% 47.4%)
LDL	0.0%	(-2.6% 1.5%)	0.0%	(-3.7% 3.8%)	0.0%	(-17.3% 16.4%)
Glucose	**8.1%****	**(2.2% 19.0%)**	**5.5%**	**(0.7% 18.2%)**	7.4%	(-82.9% 127.0%)
C-reactive protein	**9.5%****	**(3.2% 24.3%)**	1.7%	(-2.2% 7.7%)	-0.8%	(-37.7% 35.0%)

Supplementary Table 4. Average mediated proportions for DNA methylation and clinical mediators on the relationship between education attainment and all-cause mortality, NHANES 1999-2002 (n=2,402). Bold indicates statistical significance (p-value<0.05), and bold with two asterisks indicates significance passed Bonferroni adjustment (p-value<0.0021).

	**Model 1. Less than high school (N=1,096) vs. College degree or higher (N= 370)**		**Model 2. High school graduate/GED (N=496) vs. College degree or higher (N= 370)**		**Model 3. Some college or associates degree (N=440) vs. College degree or higher (N= 370)**	
	**Average mediated proportion**	**(95% CI)**	**Average mediated proportion**	**(95% CI)**	**Average mediated proportion**	**(95% CI)**
**Chronological age clocks**						
Hannum	1.6%	(-0.0% 4.5%)	**4.4%**	**(0.3% 13.4%)**	0.5%	(-6.9% 7.7%)
Hovarth	-0.1%	(-1.7% 1.3%)	-1.7%	(-8.9% 1.3%)	**-4.1%**	**(-14.0%- 0.2%)**
Weidner	-0.3%	(-3.1% 1.3%)	0.1%	(-3.0% 3.7%)	-0.9%	(-7.8% 1.6%)
Lin	-0.2%	(-2.1% 1.2%)	-1.2%	(-5.5% 1.0%)	-0.5%	(-5.4% 3.1%)
Vidal-Bralo	-0.7%	(-3.4% 0.5%)	-0.3%	(-5.0% 1.6%)	-0.7%	(-5.8% 2.6%)
SkinBlood	0.3%	(-1.0% 2.2%)	0.9%	(-1.2% 5.7%)	0.0%	(-3.4% 2.7%)
Zhang	0.5%	(-0.7% 2.6%)	0.2%	(-1.7% 3.1%)	-0.1%	(-5.5% 3.2%)
**Physiological functioning clocks**						
PhenoAge	**6.5%****	**(2.4% 13.7%)**	2.9%	(-1.3% 12.2%)	3.1%	(-5.0% 12.4%)
GrimAge	**23.3%****	**(14.2% 36.6%)**	**18.7%****	**(8.1% 41.7%)**	**31.8%****	**(17.8% 57.5%)**
GrimAge2	**27.6%****	**(16.4% 44.9%)**	**21.4%****	**(9.9% 48.5%)**	**32.4%****	**(17.2% 64.2%)**
**Pace of aging clock**						
DunedinPoAm	**13.6%****	**(6.7% 23.6%)**	**11.4%****	**(2.8% 27.6%)**	**22.0%****	**(11.0% 38.0%)**
**Clocks trained on other aging biomarkers**						
Yang	-1.0%	(-4.4% 1.9%)	1.2%	(-1.5% 6.0%)	0.3%	(-3.9% 4.5%)
Telomere	**5.3%**	**(1.7% 10.1%)**	**5.7%****	**(1.9% 13.1%)**	3.5%	(-0.9% 10.9%)
**Behavioral/Lifestyle measures**						
Sedentary behavior	**12.9%****	**(5.9% 23.2%)**	**21.2%**	**(9.4% 44.9%)**	6.2%	(-0.4% 17.7%)
Healthy eating index	1.8%	(-6.5% 14.1%)	1.3%	(-15.4% 14.5%)	3.7%	(-6.3% 14.6%)
Smoking pack years	**10.6%****	**(4.8% 18.3%)**	**10.4%**	**(2.6% 27.3%)**	**16.2%****	**(7.3% 35.2%)**
Alcohol drinker	1.9%	(-2.5% 7.1%)	**6.6%**	**(1.0% 19.2%)**	2.4%	(-1.7% 10.6%)
**Anthropometric measures**						
Waist to thigh ratio	**5.6%**	**(1.6% 12.6%)**	3.0%	(-4.1% 12.0%)	4.0%	(-3.4% 14.6%)
BMI	0.4%	(-3.8% 6.3%)	3.0%	(-2.7% 13.0%)	4.5%	(-7.5% 23.6%)
**Clinical used blood Biomarkers**						
Total cholesterol	0.0%	(-1.4% 1.6%)	-0.4%	(-15.5% 8.9%)	-3.4%	(-16.2% 2.8%)
HDL	0.1%	(-5.8% 5.9%)	0.3%	(-4.7% 5.4%)	0.0%	(-3.9% 3.9%)
LDL	-0.1%	(-2.3% 1.2%)	0.4%	(-13.7% 11.5%)	-3.9%	(-15.4% 0.7%)
Glucose	2.3%	(-0.2% 6.5%)	1.3%	(-2.0% 6.4%)	-1.8%	(-12.1% 3.7%)
C-reactive protein	**2.0%**	**(0.5% 4.5%)**	0.6%	(-2.7% 4.4%)	0.7%	(-3.7% 5.9%)

Supplementary Table 5. Average mediated proportions for DNA methylation and clinical mediators on the relationship between household income and all-cause mortality, NHANES 1999-2002 (n=2,402). Bold indicates statistical significance (p-value<0.05), and bold with two asterisks indicates significance passed Bonferroni adjustment (p-value<0.0021).

	Model 1. PIR<1 (N=372) vs. PIR 5+ (N= 399)		Model 2. PIR 1-2 (N=707) vs. PIR 5+ (N= 399)		Model 3. PIR 2-5 (N=923) vs. PIR 5+ (N= 399)	
	Average mediated proportion	(95% CI)	Average mediated proportion	(95% CI)	Average mediated proportion	(95% CI)
**Chronological age clocks**						
Hannum	3.0%	(-0.5% 8.2%)	2.6%	(-0.8% 6.5%)	0.3%	(-7.1% 6.6%)
Hovarth	-0.1%	(-2.6% 1.6%)	-0.1%	(-3.7% 2.9%)	-0.6%	(-5.5% 2.5%)
Weidner	0.2%	(-1.5% 3.1%)	0.5%	(-1.6% 2.9%)	0.0%	(-1.8% 1.7%)
Lin	0.2%	(-1.5% 2.7%)	-0.7%	(-3.0% 0.6%)	-1.1%	(-6.0% 1.5%)
Vidal-Bralo	-0.3%	(-3.1% 0.9%)	0.6%	(-1.9% 3.4%)	-0.3%	(-3.6% 1.9%)
SkinBlood	-0.2%	(-3.7% 1.7%)	0.0%	(-1.1% 1.2%)	-1.2%	(-5.1% 1.7%)
Zhang	-0.4%	(-2.9% 1.0%)	-0.5%	(-2.2% 0.7%)	-1.0%	(-5.3% 1.1%)
**Physiological functioning clocks**						
PhenoAge	**5.1%**	**(0.3% 13.4%)**	**8.3%**	**(2.5% 16.5%)**	4.0%	(-0.8% 15.3%)
GrimAge	**32.1%****	**(20.1% 50.0%)**	**24.7%****	**(14.5% 41.4%)**	**22.6%****	**(9.6% 53.5%)**
GrimAge2	**36.6%****	**(22.2% 57.8%)**	**29.2%****	**(17.4% 50.8%)**	**24.9%****	**(10.6% 59.5%)**
**Pace of aging clock**						
DunedinPoAm	**19.5%****	**(9.8% 35.7%)**	**14.1%****	**(6.6% 29.0%)**	**15.0%**	**(4.1% 40.5%)**
**Clocks trained on other aging biomarkers**						
Yang	0.1%	(-4.4% 5.1%)	1.0%	(-1.1% 4.8%)	0.0%	(-4.0% 1.9%)
Telomere	1.8%	(-1.2% 7.5%)	**5.4%****	**(1.9% 10.3%)**	**3.7%**	**(0.3% 11.4%)**
**Behavioral/Lifestyle measures**						
Sedentary behavior	**21.0%**	**(5.2% 39.7%)**	**12.9%**	**(4.2% 31.0%)**	**14.0%**	**(1.4% 48.4%)**
Healthy eating index	4.0%	(-10.9% 16.8%)	1.9%	(-3.7% 9.5%)	2.9%	(-3.5% 18.1%)
Smoking pack years	**11.5%****	**(5.1% 19.8%)**	**10.9%****	**(3.6% 27.9%)**	**12.7%****	**(3.8% 31.0%)**
Alcohol drinker	-3.7%	(-17.5% 7.4%)	2.1%	(-8.5% 13.6%)	**6.0%**	**(0.3% 16.6%)**
**Anthropometric measures**						
Waist to thigh ratio	5.1%	(-1.1% 12.5%)	5.8%	(-1.7% 15.3%)	8.1%	(-0.0% 21.3%)
BMI	-0.5%	(-11.2% 8.2%)	-0.4%	(-11.5% 6.0%)	2.6%	(-10.4% 17.1%)
**Clinical used blood Biomarkers**						
Total cholesterol	0.0%	(-3.6% 3.0%)	0.3%	(-1.3% 3.5%)	-0.4%	(-5.8% 4.5%)
HDL	-4.0%	(-16.7% 5.2%)	0.6%	(-7.1% 7.4%)	0.1%	(-6.9% 7.6%)
LDL	-1.7%	(-7.9% 1.9%)	0.2%	(-2.0% 3.7%)	-1.6%	(-9.7% 4.3%)
Glucose	**6.2%**	**(1.1% 14.8%)**	1.2%	(-2.5% 6.8%)	4.5%	(-0.1% 12.9%)
C-reactive protein	**2.0%**	**(0.3% 5.1%)**	**4.9%**	**(1.3% 10.0%)**	2.1%	(-0.3% 8.0%)
