# Supplementary material for: The mediating role of DNA methylation clocks in associations of race, ethnicity, education, income, and occupation with mortality: findings from NHANES 1999-2002
Source: Aging (Albany NY). 2026 May 8;18(1):466–512. doi: 10.18632/aging.206377 (PMC13632862; doi:10.18632/aging.206377)
Supplement: Supplementary file 3 [file aging-18-1-206377-s006.pdf]

Supplementary Table 7. Average mediated proportions for DNA methylation and clinical mediators on the relationship between race and ethnicity and cardiovascular-specific mortality, NHANES 1999-2002 (n=2,402). Bold indicates statistical significance (p-value<0.05), and bold with two asterisks indicates significance passed Bonferroni adjustment (p-value<0.0021).

	Model 1. Black (N= 516) vs. White (N= 943)		Model 2. Hispanic (N=860) vs. White (N= 943)		Model 3. Other races (N=83) vs. White (N= 943)	
	Average mediated proportion	(95% CI)	Average mediated proportion	(95% CI)	Average mediated proportion	(95% CI)
**Chronological age clocks**						
Hannum	-3.6%	(-15.4% 14.2%)	1.2%	(-74.1% 88.9%)	-0.1%	(-11.3% 8.8%)
Hovarth	-0.7%	(-5.6% 1.9%)	0.1%	(-37.3% 30.2%)	0.0%	(-4.8% 4.5%)
Weidner	0.0%	(-4.9% 6.5%)	-0.4%	(-77.3% 71.0%)	-0.3%	(-24.7% 19.3%)
Lin	-0.1%	(-4.7% 4.8%)	-0.3%	(-59.8% 60.2%)	-0.1%	(-14.0% 12.6%)
Vidal-Bralo	-2.6%	(-17.9% 11.5%)	-0.9%	(-153.0% 132.5%)	0.3%	(-22.6% 16.8%)
SkinBlood	-2.0%	(-9.7% 6.7%)	0.1%	(-20.4% 30.9%)	-0.1%	(-9.2% 7.9%)
Zhang	-4.0%	(-16.8% 7.4%)	-0.2%	(-24.3% 22.5%)	0.3%	(-9.3% 9.8%)
**Physiological functioning clocks**						
PhenoAge	0.5%	(-3.2% 7.3%)	-1.4%	(-94.0% 86.9%)	0.7%	(-18.0% 20.6%)
GrimAge	**17.2%****	**(6.6% 55.5%)**	3.7%	(-207.4% 199.3%)	-2.9%	(-38.8% 21.0%)
GrimAge2	**22.6%****	**(8.7% 63.4%)**	15.5%	(-508.3% 538.3%)	-5.7%	(-94.2% 36.5%)
**Pace of aging clock**						
DunedinPoAm	**10.1%**	**(2.0% 42.8%)**	9.9%	(-410.7% 444.4%)	-5.7%	(-50.2% 12.9%)
**Clocks trained on other aging biomarkers**						
Yang	5.9%	(-31.4% 36.2%)	4.8%	(-224.1% 287.9%)	-3.8%	(-62.4% 30.8%)
Telomere	-15.5%	(-43.2% 12.9%)	0.3%	(-54.5% 68.6%)	-1.5%	(-32.9% 16.3%)
**Behavioral/Lifestyle measures**						
Sedentary behavior	**25.9%****	**(11.2% 91.9%)**	20.4%	(-635.6% 610.1%)	-11.2%	(-122.4% 29.5%)
Healthy eating index	-2.6%	(-15.0% 2.5%)	0.0%	(-49.6% 42.9%)	0.0%	(-8.5% 7.0%)
Smoking pack years	**-5.1%**	**(-12.5% - 0.2%)**	-4.8%	(-116.5% 152.5%)	1.6%	(-11.1% 20.6%)
Alcohol drinker	**11.9%**	**(0.7% 43.2%)**	5.7%	(-218.4% 219.6%)	-14.7%	(-102.4% 75.8%)
**Anthropometric measures**						
Waist to thigh ratio	**-18.9%**	**(-66.6% - 3.8%)**	10.2%	(-260.1% 343.1%)	-4.1%	(-81.8% 48.0%)
BMI	1.3%	(-11.6% 32.6%)	0.4%	(-116.9% 202.3%)	0.0%	(-17.2% 14.5%)
**Clinical used blood Biomarkers**						
Total cholesterol	0.2%	(-6.3% 5.8%)	0.0%	(-31.6% 34.7%)	0.1%	(-11.1% 10.8%)
HDL	1.5%	(-5.9% 10.3%)	2.1%	(-245.2% 198.8%)	0.4%	(-20.6% 23.6%)
LDL	0.2%	(-2.5% 4.6%)	-0.1%	(-38.3% 43.6%)	-0.1%	(-9.9% 7.6%)
Glucose	**6.8%**	**(1.0% 24.0%)**	5.3%	(-161.3% 119.6%)	-4.0%	(-41.0% 29.2%)
C-reactive protein	0.7%	(-8.8% 7.3%)	0.1%	(-11.9% 17.1%)	0.0%	(-3.2% 3.4%)

Supplementary Table 8. Average mediated proportions for DNA methylation and clinical mediators on the relationship between education attainment and cardiovascular-specific mortality, NHANES 1999-2002 (n=2,402). Bold indicates statistical significance (p-value<0.05), and bold with two asterisks indicates significance passed Bonferroni adjustment (p-value<0.0021).

	Model 1. Less than high school (N=1,096) vs. College degree or higher (N= 370)			Model 2. High school graduate/GED (N=496) vs. College degree or higher (N= 370)			Model 3. Some college or associates degree (N=440) vs. College degree or higher (N= 370)		
	Average mediated proption	(95% CI)		Average mediated proption	(95% CI)		Average mediated proption	(95% CI)	
**Chronological age clocks**									
Hannum	-1.7%	(-57.7%	82.6%)	1.1%	(-76.1%	87.7%)	0.1%	(-38.7%	36.1%)
Hovarth	0.0%	(-8.2%	7.5%)	0.1%	(-29.6%	31.4%)	1.5%	(-46.9%	38.3%)
Weidner	-0.7%	(-29.4%	35.3%)	0.0%	(-64.8%	29.6%)	-0.1%	(-29.3%	38.6%)
Lin	0.0%	(-17.6%	7.5%)	-0.1%	(-28.6%	44.2%)	0.8%	(-55.9%	32.3%)
Vidal-Bralo	-0.1%	(-15.7%	14.7%)	0.0%	(-41.3%	36.6%)	-0.4%	(-22.4%	15.8%)
SkinBlood	-0.6%	(-35.0%	12.4%)	2.1%	(-144.9%	96.5%)	0.5%	(-16.5%	26.4%)
Zhang	0.0%	(-8.3%	8.8%)	0.2%	(-22.0%	43.2%)	0.7%	(-20.6%	23.7%)
**Physiological functioning clocks**									
PhenoAge	1.6%	(-36.1%	52.0%)	-0.5%	(-117.6%	101.0%)	-1.7%	(-86.0%	81.4%)
GrimAge	51.0%	(-335.8%	531.6%)	23.8%	(-686.2%	733.5%)	12.0%	(-168.5%	212.3%)
GrimAge2	60.9%	(-255.1%	610.7%)	19.5%	(-440.8%	634.5%)	15.7%	(-281.5%	232.3%)
**Pace of aging clock**									
DunedinPoAm	18.7%	(-248.0%	269.5%)	3.7%	(-177.3%	194.5%)	14.1%	(-220.8%	268.1%)
**Clocks trained on other aging biomarkers**									
Yang	6.2%	(-48.1%	72.3%)	-0.6%	(-112.2%	115.6%)	1.1%	(-42.9%	37.1%)
Telomere	5.8%	(-46.7%	50.9%)	0.6%	(-130.4%	71.3%)	-0.7%	(-30.8%	29.2%)
**Behavioral/Lifestyle measures**									
Sedentary behavior	28.7%	(-629.4%	548.1%)	53.8%	(-1,118.9%	1312.3%)	13.1%	(-292.9%	196.2%)
Healthy eating index	-14.5%	(-153.0%	140.7%)	-8.2%	(-330.4%	367.9%)	-3.3%	(-86.7%	63.5%)
Smoking pack years	22.7%	(-301.1%	272.8%)	12.0%	(-302.9%	408.7%)	-8.5%	(-99.0%	144.2%)
Alcohol drinker	5.3%	(-110.1%	139.0%)	9.8%	(-517.8%	332.7%)	5.8%	(-87.7%	165.8%)
**Anthropometric measures**									
Waist to thigh ratio	7.4%	(-106.0%	105.0%)	3.1%	(-129.6%	133.4%)	3.1%	(-55.4%	81.2%)
BMI	-2.0%	(-70.5%	115.5%)	-2.5%	(-138.6%	143.0%)	12.0%	(-125.7%	188.6%)
**Clinical used blood Biomarkers**									
Total cholesterol	0.1%	(-31.9%	41.4%)	-4.2%	(-174.4%	210.4%)	2.5%	(-124.7%	118.7%)
HDL	-4.9%	(-95.7%	102.3%)	-0.6%	(-81.2%	80.1%)	0.6%	(-40.4%	42.5%)
LDL	-0.9%	(-75.5%	79.9%)	-0.5%	(-194.1%	201.6%)	-0.2%	(-94.4%	107.8%)
Glucose	12.4%	(-218.9%	219.7%)	0.1%	(-62.0%	73.4%)	-0.3%	(-51.9%	52.0%)
C-reactive protein	1.0%	(-28.0%	35.6%)	-0.6%	(-51.5%	59.1%)	0.1%	(-8.8%	8.4%)

Supplementary Table 9. Average mediated proportions for DNA methylation and clinical mediators on the relationship between household income and cardiovascular-specific mortality, NHANES 1999-2002 (n=2,402). Bold indicates statistical significance (p-value<0.05), and bold with two asterisks indicates significance passed Bonferroni adjustment (p-value<0.0021).

	Model 1. PIR<1 (N=373) vs. PIR 5+ (N= 399)		Model 2. PIR 1-2 (N=707) vs. PIR 5+ (N= 399)		Model 3. PIR 2-5 (N=923) vs. PIR 5+ (N= 399)	
	Average mediated propotion	(95% CI)	Average mediated proption	(95% CI)	Average mediated proption	(95% CI)
**Chronological age clocks**						
Hannum	0.5%	(-6.0% 6.5%)	0.0%	(-13.2% 7.1%)	0.0%	(-22.0% 21.7%)
Hovarth	0.7%	(-2.9% 6.2%)	0.0%	(-5.7% 4.9%)	-0.1%	(-15.7% 11.7%)
Weidner	0.0%	(-6.4% 5.1%)	-0.8%	(-29.3% 9.6%)	0.0%	(-14.1% 15.8%)
Lin	-0.2%	(-7.3% 7.9%)	0.7%	(-6.1% 11.4%)	-0.1%	(-19.4% 18.4%)
Vidal-Bralo	-0.1%	(-4.6% 2.6%)	-0.4%	(-11.4% 5.3%)	-0.1%	(-29.3% 16.9%)
SkinBlood	0.2%	(-6.7% 8.3%)	0.0%	(-4.9% 6.6%)	-0.8%	(-37.4% 33.5%)
Zhang	0.4%	(-2.2% 5.7%)	0.2%	(-6.0% 6.5%)	-1.3%	(-41.5% 21.6%)
**Physiological functioning clocks**						
PhenoAge	-3.7%	(-20.1% 2.1%)	-1.3%	(-30.4% 17.2%)	-1.1%	(-47.3% 50.9%)
GrimAge	**23.4%****	**(11.2% 49.9%)**	27.3%	(-36.2% 122.2%)	7.9%	(-152.7%183.1%)
GrimAge2	**25.7%****	**(11.2% 61.9%)**	33.1%	(-62.3% 201.6%)	9.1%	(-289.5%225.5%)
**Pace of aging clock**						
DunedinPoAm	15.7%	(-1.1% 47.1%)	14.6%	(-32.5% 98.0%)	5.4%	(-223.1%197.6%)
**Clocks trained on other aging biomarkers**						
Yang	0.3%	(-12.1% 11.5%)	1.5%	(-22.2% 38.4%)	0.0%	(-55.2% 45.3%)
Telomere	-1.9%	(-21.0% 4.4%)	-2.9%	(-48.2% 24.3%)	1.3%	(-31.5% 55.3%)
**Behavioral/Lifestyle measures**						
Sedentary behavior	12.6%	(-33.4% 70.4%)	40.5%	(-199.0% 301.0%)	22.1%	(-534.7%390.8%)
Healthy eating index	6.7%	(-12.1% 26.4%)	-9.4%	(-77.8% 13.8%)	-3.9%	(-71.0% 104.5%)
Smoking pack years	3.3%	(-3.5% 13.8%)	3.1%	(-21.3% 29.2%)	4.7%	(-101.5%149.9%)
Alcohol drinker	8.9%	(-8.4% 38.4%)	-1.2%	(-67.2% 53.8%)	16.7%	(-301.7%310.6%)
**Anthropometric measures**						
Waist to thigh ratio	14.9%	(-0.4% 50.4%)	11.2%	(-10.5% 68.5%)	4.7%	(-206.7%177.7%)
BMI	0.0%	(-15.5% 14.0%)	-15.3%	(-77.4% 47.2%)	22.3%	(-192.0%230.1%)
**Clinical used blood Biomarkers**						
Total cholesterol	-0.1%	(-11.2% 9.9%)	1.1%	(-14.0% 27.1%)	-0.2%	(-94.6% 90.6%)
HDL	3.8%	(-10.5% 30.8%)	-3.3%	(-93.2% 41.9%)	0.5%	(-98.7% 115.3%)
LDL	-2.9%	(-21.4% 8.0%)	0.2%	(-26.7% 19.3%)	-0.5%	(-112.7% 93.4%)
Glucose	**6.7%**	**(1.3% 27.6%)**	1.8%	(-13.5% 31.9%)	3.3%	(-96.1% 75.8%)
C-reactive protein	1.7%	(-0.8% 9.8%)	1.9%	(-11.0% 17.7%)	-0.5%	(-22.6% 21.0%)
