# Supplementary material for: The mediating role of DNA methylation clocks in associations of race, ethnicity, education, income, and occupation with mortality: findings from NHANES 1999-2002
Source: Aging (Albany NY). 2026 May 8;18(1):466–512. doi: 10.18632/aging.206377 (PMC13632862; doi:10.18632/aging.206377)
Supplement: Supplementary file 4 [file aging-18-1-206377-s008.pdf]

Supplementary Table 11. Average mediated proportions for DNA methylation and clinical mediators on the relationship between race and ethnicity and cancer-specific mortality, NHANES 1999-2002 (n=2,402). Bold indicates statistical significance (p-value<0.05), and bold with two asterisks indicates significance passed Bonferroni adjustment (p-value<0.0021).

	Model 1. Black (N= 516) vs. White (N= 943)		Model 2. Hispanic (N=860) vs. White (N= 943)		Model 3. Other races (N=83) vs. White (N= 943)	
	Average mediated proportion	(95% CI)	Average mediated proportion	(95% CI)	Average mediated proportion	(95% CI)
**Chronological age clocks**						
Hannum	-30.2%	(-300.1% 141.6%)	3.8%	(-127.4% 179.2%)	0.3%	(-111.4% 149.8%)
Hovarth	-10.9%	(-165.3% 56.1%)	-2.0%	(-159.6% 170.6%)	0.0%	(-64.9% 64.9%)
Weidner	-2.8%	(-31.6% 15.9%)	-0.8%	(-106.8% 78.3%)	-1.1%	(-91.5% 88.0%)
Lin	-3.6%	(-37.0% 40.7%)	-0.7%	(-80.3% 66.1%)	-0.5%	(-68.1% 68.3%)
Vidal-Bralo	-28.6%	(-261.0% 197.8%)	-11.2%	(-395.7% 316.8%)	-4.9%	(-324.3% 269.6%)
SkinBlood	-5.2%	(-53.5% 47.5%)	0.0%	(-17.9% 17.5%)	0.2%	(-37.5% 38.5%)
Zhang	-7.1%	(-70.1% 17.6%)	-0.1%	(-17.1% 20.4%)	-0.1%	(-32.7% 34.1%)
**Physiological functioning clocks**						
PhenoAge	-8.4%	(-76.0% 84.3%)	1.5%	(-189.7% 128.3%)	0.6%	(-158.6% 118.8%)
GrimAge	40.1%	(-256.6% 455.9%)	4.4%	(-272.9% 360.0%)	2.4%	(-352.1% 187.8%)
GrimAge2	53.6%	(-307.1% 765.2%)	23.4%	(-593.5% 795.9%)	7.0%	(-178.9% 261.1%)
**Pace of aging clock**						
DunedinPoAm	21.5%	(-258.2% 261.4%)	17.9%	(-465.7% 619.5%)	9.8%	(-286.4% 322.0%)
**Clocks trained on other aging biomarkers**						
Yang	2.1%	(-126.5% 107.1%)	-4.7%	(-206.1% 138.8%)	-3.3%	(-105.5% 174.4%)
Telomere	-54.2%	(-665.0% 414.6%)	2.0%	(-144.2% 203.1%)	-2.4%	(-189.6% 220.8%)
**Behavioral/Lifestyle measures**						
Sedentary behavior	9.2%	(-116.7% 173.0%)	3.7%	(-225.0% 218.1%)	1.7%	(-98.0% 143.0%)
Healthy eating index	25.8%	(-130.7% 268.6%)	7.0%	(-271.6% 278.2%)	0.0%	(-140.0% 124.9%)
Smoking pack years	-19.5%	(-150.5% 77.0%)	-8.9%	(-275.0% 269.8%)	-2.3%	(-179.2% 128.6%)
Alcohol drinker	-5.6%	(-118.9% 67.5%)	-0.9%	(-147.8% 168.6%)	1.8%	(-216.9% 219.7%)
**Anthropometric measures**						
Waist to thigh ratio	-18.5%	(-181.3% 80.9%)	1.1%	(-252.0% 137.5%)	0.7%	(-101.5% 116.1%)
BMI	0.4%	(-29.9% 44.9%)	-0.2%	(-54.7% 47.3%)	0.0%	(-21.3% 27.4%)
**Clinical used blood Biomarkers**						
Total cholesterol	-3.1%	(-57.4% 23.2%)	-0.1%	(-38.9% 36.1%)	-0.1%	(-57.5% 64.6%)
HDL	0.8%	(-15.6% 27.0%)	2.8%	(-153.4% 138.9%)	-0.5%	(-49.4% 36.6%)
LDL	-1.3%	(-30.5% 21.8%)	0.2%	(-55.5% 55.5%)	0.0%	(-71.2% 63.7%)
Glucose	-10.8%	(-184.9% 120.3%)	0.0%	(-106.7% 173.2%)	1.1%	(-89.4% 101.3%)
C-reactive protein	7.1%	(-61.0% 73.4%)	0.2%	(-37.1% 30.0%)	0.2%	(-34.6% 27.8%)

Supplementary Table 12. Average mediated proportions for DNA methylation and clinical mediators on the relationship between education attainment and cancer-specific mortality, NHANES 1999-2002 (n=2,402). Bold indicates statistical significance (p-value<0.05), and bold with two asterisks indicates significance passed Bonferroni adjustment (p-value<0.0021).

	Model 1. Less than high school (N=1,096) vs. College degree or higher (N= 370)		Model 2. High school graduate/GED (N=496) vs. College degree or higher (N= 370)		Model 3. Some college or associates degree (N=440) vs. College degree or higher (N= 370)	
	**Average mediated proption**	**(95% CI)**	**Average mediated proption**	**(95% CI)**	**Average mediated proption**	**(95% CI)**
**Chronological age clocks**						
Hannum	1.0%	(-1.3% 5.6%)	3.8%	(-0.1% 14.5%)	0.5%	(-5.2% 5.5%)
Hovarth	-0.1%	(-3.4% 1.9%)	-1.9%	(-8.9% 1.0%)	-3.4%	(-12.1% 0.1%)
Weidner	0.9%	(-1.9% 6.9%)	0.0%	(-2.5% 2.9%)	-1.3%	(-7.8% 1.6%)
Lin	0.1%	(-1.5% 2.4%)	-1.2%	(-6.2% 1.3%)	-1.2%	(-5.7% 0.9%)
Vidal-Bralo	-0.5%	(-4.4% 1.5%)	-0.3%	(-5.5% 2.5%)	-0.5%	(-6.6% 2.9%)
SkinBlood	-0.7%	(-4.2% 0.4%)	0.2%	(-5.5% 4.3%)	-0.1%	(-2.3% 2.2%)
Zhang	0.0%	(-1.3% 0.8%)	0.0%	(-2.0% 2.1%)	0.0%	(-3.4% 2.9%)
**Physiological functioning clocks**						
PhenoAge	2.2%	(-2.5% 12.1%)	2.5%	(-1.7% 13.2%)	1.2%	(-2.9% 8.4%)
GrimAge	**20.3%**	**(5.4% 56.1%)**	**9.0%****	**(2.7% 30.1%)**	**13.3%**	**(2.8% 36.0%)**
GrimAge2	**20.0%**	**(3.5% 48.9%)**	**10.2%**	**(3.8% 30.4%)**	12.1%	(-0.9% 34.4%)
**Pace of aging clock**						
DunedinPoAm	9.7%	(-1.5% 37.6%)	**6.6%**	**(0.7% 25.6%)**	6.5%	(-3.4% 20.9%)
**Clocks trained on other aging biomarkers**						
Yang	-1.7%	(-18.8% 5.5%)	0.4%	(-5.0% 4.7%)	0.1%	(-5.1% 7.3%)
Telomere	2.8%	(-2.3% 12.7%)	2.9%	(-3.7% 11.5%)	1.3%	(-1.6% 6.6%)
**Behavioral/Lifestyle measures**						
Sedentary behavior	2.0%	(-12.6% 27.5%)	11.4%	(-1.4% 38.2%)	-0.8%	(-13.2% 8.0%)
Healthy eating index	-0.8%	(-18.3% 45.1%)	**18.8%**	**(3.6% 55.4%)**	**11.0%**	**(2.0% 26.0%)**
Smoking pack years	6.3%	(-0.6% 27.3%)	**7.8%**	**(2.1% 29.6%)**	**9.9%****	**(4.0% 25.5%)**
Alcohol drinker	-6.5%	(-32.9% 1.3%)	-5.4%	(-35.1% 1.8%)	-0.5%	(-10.3% 4.6%)
**Anthropometric measures**						
Waist to thigh ratio	-1.5%	(-22.3% 8.2%)	1.8%	(-2.9% 10.0%)	0.3%	(-3.4% 5.5%)
BMI	-8.8%	(-46.6% 2.0%)	-0.5%	(-6.3% 5.7%)	3.6%	(-4.0% 11.6%)
**Clinical used blood Biomarkers**						
Total cholesterol	0.0%	(-5.9% 3.9%)	**7.2%**	**(1.2% 27.1%)**	-5.6%	(-24.9% 0.9%)
HDL	-4.2%	(-22.0% 1.7%)	-1.2%	(-13.0% 1.5%)	0.7%	(-4.2% 9.2%)
LDL	0.0%	(-6.4% 5.1%)	**8.4%**	**(1.4% 27.5%)**	-3.9%	(-16.3% 2.1%)
Glucose	-3.3%	(-20.7% 4.4%)	-1.7%	(-10.8% 2.2%)	-0.2%	(-4.7% 2.0%)
C-reactive protein	0.5%	(-1.4% 3.7%)	0.1%	(-1.5% 1.6%)	0.1%	(-1.7% 2.9%)

Supplementary Table 13. Average mediated proportions for DNA methylation and clinical mediators on the relationship between household income and cancer-specific mortality, NHANES 1999-2002 (n=2,402). Bold indicates statistical significance (p-value<0.05), and bold with two asterisks indicates significance passed Bonferroni adjustment (p-value<0.0021).

	Model 1. PIR<1 (N=373) vs. PIR 5+ (N= 399)		Model 2. PIR 1-2 (N=707) vs. PIR 5+ (N= 399)		Model 3. PIR 2-5 (N=923) vs. PIR 5+ (N= 399)	
	Average mediated proption	(95% CI)	Average mediated proption	(95% CI)	Average mediated proption	(95% CI)
**Chronological age clocks**						
Hannum	4.6%	(-22.2% 47.4%)	1.7%	(-6.5% 16.9%)	0.4%	(-53.5% 84.1%)
Hovarth	-0.1%	(-23.6% 27.5%)	0.0%	(-7.4% 5.6%)	-1.3%	(-45.4% 41.3%)
Weidner	-0.3%	(-30.3% 20.4%)	0.7%	(-11.4% 17.5%)	0.0%	(-15.2% 9.4%)
Lin	2.9%	(-12.8% 37.2%)	-0.4%	(-12.9% 7.7%)	-1.2%	(-32.1% 20.3%)
Vidal-Bralo	-2.6%	(-91.4% 42.0%)	0.2%	(-8.5% 13.0%)	-0.8%	(-50.5% 35.1%)
SkinBlood	0.7%	(-13.4% 24.9%)	0.0%	(-4.7% 4.9%)	-2.8%	(-33.1% 36.6%)
Zhang	0.1%	(-22.1% 16.1%)	0.5%	(-3.5% 9.4%)	-1.1%	(-30.6% 19.3%)
**Physiological functioning clocks**						
PhenoAge	5.0%	(-49.0% 74.1%)	7.5%	(-9.2% 56.9%)	8.0%	(-100.5% 148.4%)
GrimAge	**75.8%**	**(30.3% 325.9%)**	29.2%	(-3.0% 161.3%)	35.3%	(-479.9% 617.9%)
GrimAge2	**79.7%**	**(28.4% 415.5%)**	35.4%	(-3.4% 195.6%)	38.0%	(-388.4% 648.5%)
**Pace of aging clock**						
DunedinPoAm	**53.9%**	**(11.8% 240.9%)**	13.0%	(-13.4% 106.8%)	19.4%	(-286.6% 352.8%)
**Clocks trained on other aging biomarkers**						
Yang	-0.8%	(-38.8% 36.9%)	0.1%	(-15.6% 24.2%)	-0.2%	(-24.0% 28.9%)
Telomere	12.5%	(-40.6% 116.3%)	8.5%	(-3.8% 54.0%)	3.6%	(-43.5% 53.3%)
**Behavioral/Lifestyle measures**						
Sedentary behavior	41.3%	(-265.1% 342.8%)	-0.8%	(-65.8% 107.4%)	5.7%	(-256.2% 194.7%)
Healthy eating index	67.6%	(-528.8% 618.2%)	21.1%	(-131.8% 252.1%)	19.3%	(-444.2% 516.9%)
Smoking pack years	**31.9%**	**(11.7% 127.7%)**	23.4%	(-114.1% 171.3%)	18.5%	(-305.0% 354.0%)
Alcohol drinker	-50.3%	(-316.9% 56.6%)	-18.1%	(-178.6% 68.2%)	-4.4%	(-156.4% 165.7%)
**Anthropometric measures**						
Waist to thigh ratio	-8.0%	(-151.2% 74.2%)	6.3%	(-27.1% 79.5%)	7.0%	(-118.1% 201.9%)
BMI	-3.1%	(-116.3% 71.6%)	-10.3%	(-87.9% 38.2%)	6.0%	(-134.5% 210.4%)
**Clinical used blood Biomarkers**						
Total cholesterol	-2.2%	(-40.1% 11.2%)	-0.5%	(-21.4% 10.0%)	7.0%	(-113.5% 139.8%)
HDL	20.0%	(-61.7% 172.3%)	12.8%	(-28.2% 106.7%)	-4.2%	(-116.4% 89.8%)
LDL	-2.8%	(-56.1% 31.5%)	-0.1%	(-24.1% 15.9%)	12.8%	(-233.9% 270.1%)
Glucose	-10.6%	(-168.7% 122.5%)	0.2%	(-19.2% 30.6%)	-5.6%	(-147.4% 111.6%)
C-reactive protein	5.2%	(-12.7% 42.7%)	3.0%	(-21.3% 43.1%)	2.4%	(-74.5% 46.3%)
