# Supplementary material for: The mediating role of DNA methylation clocks in associations of race, ethnicity, education, income, and occupation with mortality: findings from NHANES 1999-2002
Source: Aging (Albany NY). 2026 May 8;18(1):466–512. doi: 10.18632/aging.206377 (PMC13632862; doi:10.18632/aging.206377)
Supplement: Supplementary file 5 [file aging-18-1-206377-s010.pdf]

Supplementary Table 15. Average mediated proportions for DNA methylation and clinical mediators on the relationship between race and ethnicity and all-cause mortality among women of NHANES 1999-2002 (n=1,172). Bold indicates statistical significance (p-value<0.05), and bold with two asterisks indicates significance passed Bonferroni adjustment (p-value<0.0021).

	Model 1. Black (N=250) vs. White (N=456)		Model 2. Hispanic (N=419) vs. White (N=456)		Model 3. Other races (N=47) vs. White (N= 456)	
	Average mediated proption	(95% CI)	Average mediated proption	(95% CI)	Average mediated proption	(95% CI)
**Chronological age clocks**						
Hannum	-44.6%	(-362.1% 237.7%)	2.0%	(-15.0% 16.5%)	3.6%	(-126.7% 116.8%)
Hovarth	-12.0%	(-125.2% 42.3%)	-7.4%	(-27.0% 0.2%)	-1.4%	(-48.8% 53.2%)
Weidner	-14.6%	(-139.0% 67.0%)	**-8.8%**	**(-26.6% - 0.3%)**	-14.9%	(-260.1% 144.7%)
Lin	-11.2%	(-112.1% 55.2%)	**-8.5%**	**(-29.3% - 0.9%)**	-5.7%	(-108.2% 101.9%)
Vidal-Bralo	-40.5%	(-440.9% 214.2%)	**-14.2%****	**(-38.7% - 4.9%)**	-17.7%	(-152.1% 263.1%)
SkinBlood	-17.3%	(-259.4% 109.7%)	-2.9%	(-16.6% 4.7%)	1.1%	(-59.2% 52.8%)
Zhang	-28.5%	(-233.7% 197.7%)	-3.5%	(-15.5% 2.1%)	-2.3%	(-81.1% 49.3%)
**Physiological functioning clocks**						
PhenoAge	-24.5%	(-274.6% 172.6%)	2.1%	(-9.8% 16.7%)	0.1%	(-84.3% 59.8%)
GrimAge	24.2%	(-117.2% 213.7%)	3.1%	(-20.3% 20.8%)	17.5%	(-114.6% 239.1%)
GrimAge2	43.2%	(-251.1% 286.3%)	**18.7%**	**(2.3% 44.1%)**	24.9%	(-119.9% 312.7%)
**Pace of aging clock**						
DunedinPoAm	18.8%	(-38.2% 170.4%)	**15.3%****	**(6.0% 34.9%)**	21.6%	(-112.6% 219.5%)
**Clocks trained on other aging biomarkers**						
Yang	9.8%	(-80.7% 151.7%)	1.8%	(-9.2% 14.3%)	-1.0%	(-97.3% 92.4%)
Telomere	-89.1%	(-656.7% 424.6%)	3.1%	(-16.4% 18.4%)	8.2%	(-179.9% 320.9%)
**Behavioral/Lifestyle measures**						
Sedentary behavior	33.1%	(-81.6% 184.1%)	**16.1%**	**(2.3% 38.9%)**	15.5%	(-171.0% 147.2%)
Healthy eating index	2.1%	(-18.7% 28.5%)	2.1%	(-3.9% 11.7%)	0.6%	(-31.6% 31.8%)
Smoking pack years	-28.4%	(-272.6% 193.4%)	**-7.0%**	**(-17.4% - 1.9%)**	-3.4%	(-62.0% 48.6%)
Alcohol drinker	30.1%	(-133.1% 195.9%)	**13.4%**	**(2.2% 40.2%)**	23.1%	(-307.2% 459.0%)
**Anthropometric measures**						
Waist to thigh ratio	-21.7%	(-166.9% 1.3%)	**14.9%**	**(3.3% 45.0%)**	7.6%	(-98.0% 103.0%)
BMI	9.0%	(-49.8% 77.3%)	1.2%	(-7.8% 10.4%)	0.9%	(-16.4% 21.1%)
**Clinical used blood Biomarkers**						
Total cholesterol	0.0%	(-12.8% 10.2%)	0.0%	(-5.4% 8.0%)	0.0%	(-23.7% 18.8%)
HDL	0.0%	(-14.9% 13.8%)	6.1%	(-8.1% 26.0%)	1.2%	(-57.2% 53.9%)
LDL	0.5%	(-15.3% 18.3%)	0.1%	(-7.3% 10.3%)	0.2%	(-40.3% 47.0%)
Glucose	25.6%	(-90.6% 216.3%)	2.2%	(-3.5% 12.4%)	6.3%	(-67.0% 91.1%)
C-reactive protein	19.9%	(-61.4% 163.7%)	0.2%	(-2.5% 3.5%)	-0.7%	(-30.5% 29.7%)

Supplementary Table 16. Average mediated proportions for DNA methylation and clinical mediators on the relationship between race and ethnicity and all-cause mortality among men of NHANES 1999-2002 (n=1,230). Bold indicates statistical significance (p-value<0.05), and bold with two asterisks indicates significance passed Bonferroni adjustment (p-value<0.0021).

	**Model 1. Black (N=266) vs. White (N=487)**		**Model 2. Hispanic (N=441) vs. White (N=487)**		**Model 3. Other races (N=36) vs. White (N=487)**	
	**Average mediated proption**	**(95% CI)**	**Average mediated proption**	**(95% CI)**	**Average mediated proption**	**(95% CI)**
**Chronological age clocks**						
Hannum	**-9.1%**	**(-19.5% - 3.1%)**	6.0%	(-197.8% 177.4%)	-3.0%	(-142.4% 172.4%)
Hovarth	-0.6%	(-6.9% 4.4%)	1.1%	(-101.8% 79.8%)	-0.3%	(-76.2% 61.8%)
Weidner	0.0%	(-2.0% 1.6%)	0.0%	(-43.4% 42.2%)	0.0%	(-26.8% 34.2%)
Lin	0.1%	(-2.9% 3.8%)	0.2%	(-71.0% 62.3%)	0.0%	(-35.2% 26.5%)
Vidal-Bralo	-3.9%	(-13.1% 1.1%)	-0.4%	(-65.3% 64.0%)	1.1%	(-45.7% 46.4%)
SkinBlood	-0.4%	(-4.7% 1.5%)	0.1%	(-58.6% 37.7%)	-0.5%	(-44.9% 31.7%)
Zhang	-3.8%	(-11.8% 0.8%)	0.2%	(-41.4% 36.7%)	0.1%	(-34.2% 35.9%)
**Physiological functioning clocks**						
PhenoAge	2.7%	(-5.7% 11.8%)	11.3%	(-366.7% 298.5%)	-6.2%	(-320.5% 143.0%)
GrimAge	**43.9%****	**(22.0% 92.0%)**	17.6%	(-937.4% 656.1%)	11.1%	(-297.6% 223.7%)
GrimAge2	**51.0%****	**(26.9% 95.3%)**	50.6%	(-848.5% 1216.9%)	7.1%	(-286.5% 245.1%)
**Pace of aging clock**						
DunedinPoAm	**29.2%****	**(12.8% 57.8%)**	38.3%	(-569.0% 753.6%)	-5.3%	(-297.3% 166.7%)
**Clocks trained on other aging biomarkers**						
Yang	5.9%	(-5.6% 19.1%)	-0.2%	(-133.1% 151.7%)	-3.0%	(-67.0% 70.6%)
Telomere	**-24.5%****	**(-46.1% - 11.7%)**	1.1%	(-103.4% 111.3%)	-3.2%	(-204.3% 111.2%)
**Behavioral/Lifestyle measures**						
Sedentary behavior	**23.7%****	**(10.3% 54.7%)**	11.2%	(-286.8% 252.6%)	-0.1%	(-113.6% 215.3%)
Healthy eating index	**9.3%**	**(1.0% 23.7%)**	-1.0%	(-116.8% 104.5%)	10.5%	(-242.3% 276.9%)
Smoking pack years	-6.7%	(-21.4% 1.9%)	-8.1%	(-320.4% 286.0%)	10.3%	(-239.3% 262.0%)
Alcohol drinker	3.7%	(-2.6% 13.4%)	0.0%	(-38.9% 49.0%)	-2.3%	(-103.0% 127.1%)
**Anthropometric measures**						
Waist to thigh ratio	**-41.6%****	**(-113.8%- 18.0%)**	0.6%	(-227.0% 290.1%)	9.1%	(-311.9% 286.9%)
BMI	-0.1%	(-7.8% 3.9%)	0.0%	(-151.1% 142.6%)	2.8%	(-124.8% 190.4%)
**Clinical used blood Biomarkers**						
Total cholesterol	-1.2%	(-13.1% 6.0%)	0.2%	(-62.6% 73.1%)	1.7%	(-117.7% 105.1%)
HDL	0.8%	(-14.4% 14.5%)	0.0%	(-19.5% 31.9%)	0.1%	(-70.6% 29.3%)
LDL	0.5%	(-10.8% 9.2%)	0.6%	(-88.3% 84.2%)	1.2%	(-152.5% 137.7%)
Glucose	1.2%	(-1.0% 5.6%)	6.1%	(-146.0% 135.5%)	-1.7%	(-78.1% 68.9%)
C-reactive protein	**5.8%**	**(0.3% 18.4%)**	2.9%	(-89.1% 84.8%)	1.2%	(-37.8% 39.2%)

Supplementary Table 17. Average mediated proportions for DNA methylation and clinical mediators on the relationship between education attainment and all-cause mortality among women of NHANES 1999-2002 (n=1,172). Bold indicates statistical significance (p-value<0.05), and bold with two asterisks indicates significance passed Bonferroni adjustment (p-value<0.0021).

	Model 1. Less than high school (N=543) vs. College degree or higher (N=136)			Model 2. High school graduate/GED (N=271) vs. College degree or higher (N=136)			Model 3. Some college or associates degree (N=222) vs. College degree or higher (N=136)		
	Average mediated proption	(95% CI)		Average mediated proption	(95% CI)		Average mediated proption	(95% CI)	
**Chronological age clocks**									
Hannum	2.4%	(-1.0%	11.4%)	12.9%	(-14.4%	71.5%)	0.9%	(-70.9%	79.8%)
Hovarth	-0.1%	(-4.5%	2.7%)	-1.7%	(-36.6%	8.1%)	-8.5%	(-144.7%	150.9%)
Weidner	-0.9%	(-8.9%	1.8%)	-3.1%	(-64.0%	28.0%)	-5.2%	(-96.6%	86.8%)
Lin	-0.5%	(-7.5%	4.6%)	-1.2%	(-24.8%	13.3%)	-2.1%	(-88.4%	42.6%)
Vidal-Bralo	-2.4%	(-13.8%	1.5%)	-2.3%	(-27.5%	6.4%)	-5.9%	(-199.9%	93.7%)
SkinBlood	0.7%	(-2.3%	7.8%)	3.0%	(-19.0%	43.2%)	-0.3%	(-31.1%	20.0%)
Zhang	1.2%	(-3.4%	9.4%)	1.8%	(-6.7%	32.5%)	-0.8%	(-71.7%	41.7%)
**Physiological functioning clocks**									
PhenoAge	**6.7%**	**(0.3%**	**26.2%)**	5.0%	(-19.2%	45.7%)	1.8%	(-88.1%	114.6%)
GrimAge	**19.7%****	**(7.7%**	**45.4%)**	17.6%	(-4.3%	129.4%)	25.6%	(-216.3%	251.6%)
GrimAge2	**26.5%****	**(11.8%**	**63.2%)**	19.2%	(-17.5%	135.0%)	31.8%	(-200.3%	337.2%)
**Pace of aging clock**									
DunedinPoAm	**11.0%****	**(3.3%**	**26.6%)**	10.5%	(-5.7%	54.5%)	15.7%	(-175.7%	152.1%)
**Clocks trained on other aging biomarkers**									
Yang	-1.8%	(-7.1%	2.2%)	1.6%	(-10.3%	28.3%)	0.7%	(-23.2%	20.0%)
Telomere	**7.1%**	**(1.4%**	**19.5%)**	5.1%	(-9.1%	45.8%)	7.7%	(-71.4%	131.3%)
**Behavioral/Lifestyle measures**									
Sedentary behavior	**20.1%**	**(4.3%**	**70.3%)**	16.9%	(-2.6%	92.7%)	3.5%	(-73.9%	90.7%)
Healthy eating index	0.4%	(-13.1%	19.1%)	1.0%	(-65.6%	56.7%)	0.4%	(-71.5%	73.1%)
Smoking pack years	**7.6%**	**(1.0%**	**19.3%)**	5.2%	(-17.5%	65.9%)	6.4%	(-89.5%	86.0%)
Alcohol drinker	3.7%	(-8.9%	16.8%)	16.3%	(-6.0%	75.6%)	-0.9%	(-38.1%	24.7%)
**Anthropometric measures**									
Waist to thigh ratio	4.8%	(-1.0%	23.4%)	-0.6%	(-26.2%	19.5%)	-1.7%	(-76.0%	36.9%)
BMI	-0.7%	(-12.9%	12.8%)	5.6%	(-3.1%	32.8%)	0.8%	(-32.3%	33.2%)
**Clinical used blood Biomarkers**									
Total cholesterol	0.0%	(-6.6%	4.9%)	-5.2%	(-59.8%	42.9%)	-2.2%	(-92.2%	53.3%)
HDL	3.0%	(-6.0%	16.8%)	0.1%	(-12.2%	13.0%)	-0.1%	(-30.5%	38.4%)
LDL	0.1%	(-9.4%	6.5%)	-5.5%	(-73.1%	25.2%)	-0.9%	(-70.8%	72.4%)
Glucose	1.4%	(-4.2%	10.5%)	-3.0%	(-51.9%	17.1%)	0.3%	(-71.7%	69.5%)
C-reactive protein	**3.2%**	**(0.5%**	**9.9%)**	6.8%	(-3.3%	33.4%)	4.0%	(-66.4%	65.8%)

Supplementary Table 18. Average mediated proportions for DNA methylation and clinical mediators on the relationship between education attainment and all-cause mortality among men of NHANES 1999-2002 (n=1,230). Bold indicates statistical significance (p-value<0.05), and bold with two asterisks indicates significance passed Bonferroni adjustment (p-value<0.0021).

	Model 1. Less than high school (N=553) vs. College degree or higher (N=234)			Model 2. High school graduate/GED (N=225) vs. College degree or higher (N=234)			Model 3. Some college or associates degree (N=218) vs. College degree or higher (N=234)		
	Average mediated proption	(95% CI)		Average mediated proption	(95% CI)		Average mediated proption	(95% CI)	
**Chronological age clocks**									
Hannum	1.2%	(-0.3%	4.1%)	1.5%	(-3.0%	8.3%)	-0.2%	(-11.2%	10.2%)
Hovarth	0.0%	(-2.3%	1.8%)	-1.9%	(-10.6%	2.2%)	-2.4%	(-13.7%	1.6%)
Weidner	0.0%	(-2.4%	2.3%)	-0.3%	(-6.2%	2.5%)	0.0%	(-4.5%	4.3%)
Lin	-0.1%	(-2.3%	0.9%)	-1.3%	(-8.3%	1.7%)	0.8%	(-5.1%	9.7%)
Vidal-Bralo	0.0%	(-1.9%	1.7%)	0.0%	(-3.0%	2.3%)	0.0%	(-4.6%	4.6%)
SkinBlood	0.1%	(-1.0%	2.1%)	0.2%	(-3.0%	4.9%)	0.0%	(-6.5%	6.4%)
Zhang	0.0%	(-1.6%	1.8%)	-1.1%	(-6.8%	2.2%)	0.3%	(-13.4%	7.5%)
**Physiological functioning clocks**									
PhenoAge	**6.7%**	**(1.6%**	**15.0%)**	1.9%	(-2.2%	10.7%)	3.6%	(-5.4%	18.0%)
GrimAge	**25.7%****	**(13.7%**	**40.3%)**	**18.7%**	**(4.3%**	**45.0%)**	**32.2%****	**(15.6%**	**80.2%)**
GrimAge2	**28.9%****	**(14.4%**	**46.8%)**	**23.3%****	**(8.5%**	**53.4%)**	**32.6%****	**(13.8%**	**82.8%)**
**Pace of aging clock**									
DunedinPoAm	**15.1%****	**(5.4%**	**28.8%)**	11.5%	(-1.1%	28.6%)	**25.0%**	**(8.9%**	**60.0%)**
**Clocks trained on other aging biomarkers**									
Yang	-0.2%	(-4.3%	3.5%)	0.2%	(-5.0%	6.2%)	0.1%	(-3.4%	4.9%)
Telomere	4.3%	(-0.1%	11.1%)	5.0%	(-2.7%	15.2%)	1.8%	(-5.2%	12.7%)
**Behavioral/Lifestyle measures**									
Sedentary behavior	6.3%	(-2.7%	16.8%)	**23.6%**	**(5.8%**	**50.3%)**	5.8%	(-2.7%	26.9%)
Healthy eating index	3.0%	(-12.2%	21.4%)	0.2%	(-21.6%	18.1%)	4.5%	(-4.7%	17.7%)
Smoking pack years	**12.9%****	**(5.2%**	**25.5%)**	**12.9%****	**(4.1%**	**29.4%)**	**22.1%****	**(7.8%**	**66.7%)**
Alcohol drinker	1.1%	(-5.2%	8.4%)	-2.2%	(-10.9%	7.9%)	**6.8%**	**(0.1%**	**29.2%)**
**Anthropometric measures**									
Waist to thigh ratio	**6.7%**	**(0.7%**	**15.8%)**	7.0%	(-1.0%	18.6%)	**13.8%**	**(0.4%**	**37.9%)**
BMI	0.1%	(-2.9%	3.6%)	-0.1%	(-12.5%	10.1%)	10.8%	(-6.3%	57.0%)
**Clinical used blood Biomarkers**									
Total cholesterol	0.0%	(-2.3%	2.0%)	2.7%	(-9.5%	14.8%)	-1.7%	(-18.1%	4.1%)
HDL	-1.1%	(-8.6%	3.2%)	-1.6%	(-12.8%	4.6%)	-1.9%	(-11.8%	14.0%)
LDL	0.0%	(-1.9%	2.1%)	2.3%	(-12.5%	15.1%)	-3.2%	(-17.6%	3.0%)
Glucose	**3.3%**	**(0.6%**	**8.5%)**	-0.4%	(-7.6%	5.8%)	0.0%	(-4.7%	2.8%)
C-reactive protein	1.2%	(-2.6%	5.8%)	-0.6%	(-6.9%	4.3%)	-0.6%	(-9.0%	4.8%)

Supplementary Table 19. Average mediated proportions for DNA methylation and clinical mediators on the relationship between household income and all-cause mortality among women of NHANES 1999-2002 (n=1,172). Bold indicates statistical significance (p-value<0.05), and bold with two asterisks indicates significance passed Bonferroni adjustment (p-value<0.0021).

	**Model 1. PIR<1 (N=202) vs. PIR 5+ (N= 175)**		**Model 2. PIR 1-2 (N=355) vs. PIR 5+ (N=175)**		**Model 3. PIR 2-5 (N=440) vs. PIR 5+ (N=175)**	
	**Average mediated proption**	**(95% CI)**	**Average mediated proption**	**(95% CI)**	**Average mediated proption**	**(95% CI)**
**Chronological age clocks**						
Hannum	4.2%	(-6.0% 18.0%)	4.6%	(-14.2% 32.9%)	0.0%	(-276.5% 257.1%)
Hovarth	-0.2%	(-8.2% 4.9%)	1.7%	(-23.4% 26.4%)	0.2%	(-82.7% 75.4%)
Weidner	0.9%	(-3.4% 7.9%)	1.9%	(-32.3% 30.7%)	0.5%	(-71.0% 102.8%)
Lin	0.9%	(-5.6% 9.9%)	-1.4%	(-17.7% 12.3%)	0.3%	(-127.4% 89.0%)
Vidal-Bralo	-2.5%	(-16.6% 1.2%)	6.8%	(-19.5% 45.8%)	-0.1%	(-93.6% 99.4%)
SkinBlood	-3.4%	(-22.5% 1.2%)	-0.7%	(-19.2% 10.8%)	-0.4%	(-65.4% 61.6%)
Zhang	-3.1%	(-21.0% 3.7%)	-2.6%	(-36.5% 10.6%)	0.1%	(-53.9% 59.4%)
**Physiological functioning clocks**						
PhenoAge	4.2%	(-2.4% 20.5%)	19.8%	(-9.0% 97.8%)	2.0%	(-287.1% 178.2%)
GrimAge	**47.7%****	**(23.5% 86.2%)**	**43.4%**	**(11.0% 265.5%)**	44.9%	(-800.3% 1444.1%)
GrimAge2	**48.1%****	**(22.9% 85.7%)**	**51.6%**	**(18.6% 221.3%)**	51.3%	(-1,069.7% 1073.6%)
**Pace of aging clock**						
DunedinPoAm	**21.4%**	**(5.5% 50.5%)**	22.4%	(-16.8% 100.7%)	19.2%	(-549.2% 536.7%)
**Clocks trained on other aging biomarkers**						
Yang	-0.1%	(-11.5% 7.7%)	0.0%	(-13.8% 6.5%)	0.2%	(-62.8% 92.7%)
Telomere	2.8%	(-2.1% 12.8%)	6.4%	(-15.3% 49.2%)	3.6%	(-211.3% 328.8%)
**Behavioral/Lifestyle measures**						
Sedentary behavior	19.5%	(-9.2% 62.4%)	27.9%	(-32.2% 214.1%)	8.5%	(-412.4% 514.2%)
Healthy eating index	7.6%	(-25.7% 39.3%)	-1.2%	(-33.9% 27.1%)	-3.4%	(-169.2% 173.0%)
Smoking pack years	**16.9%****	**(6.1% 36.0%)**	13.1%	(-15.8% 104.0%)	10.5%	(-188.8% 248.2%)
Alcohol drinker	-6.7%	(-46.2% 18.1%)	19.3%	(-34.5% 195.8%)	1.6%	(-314.5% 240.9%)
**Anthropometric measures**						
Waist to thigh ratio	1.2%	(-15.4% 13.7%)	10.2%	(-49.8% 86.7%)	2.4%	(-309.0% 287.5%)
BMI	-1.7%	(-30.0% 17.9%)	11.9%	(-27.1% 62.9%)	2.7%	(-189.6% 266.2%)
**Clinical used blood Biomarkers**						
Total cholesterol	-0.1%	(-6.3% 3.2%)	0.0%	(-11.4% 9.4%)	-1.2%	(-127.9% 117.1%)
HDL	-7.2%	(-35.8% 12.9%)	8.5%	(-33.1% 56.3%)	3.8%	(-221.5% 189.4%)
LDL	-3.3%	(-15.9% 2.9%)	0.1%	(-13.5% 13.3%)	-1.1%	(-204.1% 179.6%)
Glucose	**7.0%**	**(-0.7% 24.1%)**	4.1%	(-25.9% 65.3%)	-14.7%	(-565.7% 526.1%)
C-reactive protein	**1.4%**	**(-0.6% 5.1%)**	11.7%	(-55.4% 85.2%)	0.8%	(-141.9% 114.4%)

Supplementary Table 20. Average mediated proportions for DNA methylation and clinical mediators on the relationship between household income and all-cause mortality among men of NHANES 1999-2002 (n=1,230). Bold indicates statistical significance (p-value<0.05), and bold with two asterisks indicates significance passed Bonferroni adjustment (p-value<0.0021).

	Model 1. PIR<1 (N=171) vs. PIR 5+ (N=224)		Model 2. PIR 1-2 (N=352) vs. PIR 5+ (N=224)		Model 3. PIR 2-5 (N=483) vs. PIR 5+ (N= 224)	
	**Average mediated proption**	**(95% CI)**	**Average mediated proption**	**(95% CI)**	**Average mediated proption**	**(95% CI)**
**Chronological age clocks**						
Hannum	1.9%	(-1.0% 7.1%)	1.8%	(-0.7% 5.4%)	-1.9%	(-8.4% 1.3%)
Hovarth	0.3%	(-1.1% 3.6%)	-0.5%	(-3.1% 1.2%)	-1.2%	(-6.9% 2.3%)
Weidner	-0.3%	(-5.5% 2.5%)	0.0%	(-0.9% 1.1%)	0.0%	(-2.4% 2.4%)
Lin	0.0%	(-2.7% 3.1%)	-0.1%	(-1.9% 1.1%)	-1.1%	(-5.9% 0.9%)
Vidal-Bralo	0.1%	(-1.9% 3.0%)	-0.1%	(-2.0% 1.1%)	-0.5%	(-4.8% 1.4%)
SkinBlood	-0.5%	(-7.9% 4.5%)	0.0%	(-0.8% 0.8%)	-0.9%	(-6.2% 2.1%)
Zhang	-0.1%	(-4.2% 2.8%)	-0.2%	(-1.5% 0.5%)	-1.6%	(-7.1% 2.0%)
**Physiological functioning clocks**						
PhenoAge	**7.1%**	**(1.1% 15.9%)**	**4.1%**	**(0.7% 9.0%)**	1.5%	(-2.8% 9.7%)
GrimAge	**19.3%****	**(9.4% 32.5%)**	**18.9%****	**(10.6% 31.7%)**	12.0%	(-1.1% 34.2%)
GrimAge2	**24.4%****	**(13.3% 40.3%)**	**21.7%****	**(10.8% 35.8%)**	13.0%	(-0.5% 35.8%)
**Pace of aging clock**						
DunedinPoAm	**13.7%****	**(4.5% 27.2%)**	**11.4%****	**(4.5% 22.8%)**	9.2%	(-0.0% 27.7%)
**Clocks trained on other aging biomarkers**						
Yang	-0.3%	(-6.1% 4.6%)	3.0%	(-0.1% 8.4%)	0.0%	(-3.0% 2.5%)
Telomere	0.8%	(-4.1% 7.4%)	**4.3%**	**(0.2% 10.5%)**	1.1%	(-5.1% 8.4%)
**Behavioral/Lifestyle measures**						
Sedentary behavior	**20.6%****	**(6.4% 37.8%)**	**7.2%**	**(0.5% 15.8%)**	7.0%	(-0.4% 24.5%)
Healthy eating index	2.7%	(-5.9% 13.5%)	4.0%	(-3.7% 11.4%)	2.6%	(-4.1% 16.3%)
Smoking pack years	4.0%	(-1.4% 10.1%)	**10.6%****	**(4.4% 19.4%)**	**11.3%****	**(3.6% 27.6%)**
Alcohol drinker	2.2%	(-3.7% 9.9%)	-1.9%	(-9.1% 3.9%)	2.6%	(-8.3% 15.3%)
**Anthropometric measures**						
Waist to thigh ratio	**9.5%**	**(1.4% 20.9%)**	5.5%	(-0.4% 13.1%)	6.5%	(-0.2% 17.4%)
BMI	0.6%	(-4.7% 7.7%)	**-4.0%**	**(-9.5% - 0.8%)**	2.0%	(-3.4% 13.4%)
**Clinical used blood Biomarkers**						
Total cholesterol	0.3%	(-5.9% 8.3%)	1.0%	(-2.1% 6.9%)	-0.8%	(-7.2% 3.4%)
HDL	1.2%	(-4.2% 7.2%)	-0.8%	(-6.2% 1.8%)	0.1%	(-6.4% 7.7%)
LDL	0.0%	(-5.2% 4.2%)	0.5%	(-2.0% 4.7%)	-1.7%	(-11.1% 2.3%)
Glucose	**6.1%**	**(0.7% 14.5%)**	0.5%	(-0.6% 2.8%)	0.0%	(-1.9% 3.0%)
C-reactive protein	**4.3%**	**(0.6% 10.6%)**	2.6%	(0.0% 6.7%)	1.6%	(-0.5% 6.6%)
