# Supplementary material for: The mediating role of DNA methylation clocks in associations of race, ethnicity, education, income, and occupation with mortality: findings from NHANES 1999-2002
Source: Aging (Albany NY). 2026 May 8;18(1):466–512. doi: 10.18632/aging.206377 (PMC13632862; doi:10.18632/aging.206377)
Supplement: Supplementary file 6 [file aging-18-1-206377-s013.pdf]

Supplementary Table 23. Average mediated proportions for DNA methylation and clinical mediators on the relationship between education attainment and all-cause mortality among Black participants of NHANES 1999-2002 (n=516). Bold indicates statistical significance (p-value<0.05), and bold with two asterisks indicates significance passed Bonferroni adjustment (p-value<0.0021).

	**Model 1. Less than high school (N=259) vs. College degree or higher (N=64)**			**Model 2. High school graduate/GED (N=105) vs. College degree or higher (N=64)**			**Model 3. Some college or associates degree (N=88) vs. College degree or higher (N=64)**		
	**Average mediated proportion**	**(95% CI)**		**Average mediated proportion**	**(95% CI)**		**Average mediated proportion**	**(95% CI)**	
**Chronological age clocks**									
Hannum	**16.2%**	**(3.1%**	**50.7%)**	**45.4%**	**(8.0%**	**358.6%)**	40.3%	(-592.4%	671.8%)
Hovarth	2.4%	(-3.3%	16.7%)	18.3%	(-11.9%	77.7%)	10.4%	(-172.7%	219.2%)
Weidner	0.3%	(-3.0%	5.1%)	2.4%	(-8.0%	20.3%)	6.9%	(-120.9%	148.4%)
Lin	0.6%	(-4.0%	9.8%)	7.7%	(-14.2%	43.1%)	4.3%	(-72.6%	126.0%)
Vidal-Bralo	2.4%	(-2.8%	15.3%)	7.7%	(-22.8%	50.8%)	0.2%	(-61.9%	70.9%)
SkinBlood	3.1%	(-0.9%	14.1%)	16.0%	(-15.0%	117.6%)	2.2%	(-215.5%	177.3%)
Zhang	5.3%	(-3.4%	23.5%)	10.3%	(-19.1%	82.6%)	16.5%	(-377.7%	283.9%)
**Physiological functioning clocks**									
PhenoAge	7.6%	(-1.4%	28.6%)	16.8%	(-30.2%	120.6%)	4.8%	(-192.6%	229.6%)
GrimAge	**24.5%**	**(10.0%**	**65.6%)**	22.6%	(-21.4%	133.0%)	35.5%	(-409.4%	414.5%)
GrimAge2	**29.9%**	**(12.0%**	**86.5%)**	30.2%	(-13.6%	215.3%)	37.7%	(-344.5%	432.1%)
**Pace of aging clock**									
DunedinPoAm	**9.4%**	**(1.7%**	**28.4%)**	16.8%	(-44.2%	124.8%)	40.8%	(-397.2%	650.4%)
**Clocks trained on other aging biomarkers**									
Yang	1.6%	(-1.6%	9.5%)	5.7%	(-31.6%	66.2%)	0.5%	(-102.1%	229.6%)
Telomere	**18.0%**	**(5.2%**	**55.6%)**	55.5%	(-95.0%	323.9%)	25.4%	(-556.8%	517.2%)
**Behavioral/Lifestyle measures**									
Sedentary behavior	7.2%	(-1.8%	38.4%)	9.3%	(-4.3%	74.3%)	2.9%	(-180.9%	183.6%)
Healthy eating index	**6.7%**	**(0.4%**	**26.0%)**	5.8%	(-15.2%	78.5%)	17.1%	(-430.1%	401.6%)
Smoking pack years	**4.4%**	**(0.8%**	**18.8%)**	6.3%	(-49.7%	123.3%)	0.9%	(-140.8%	162.7%)
Alcohol drinker	0.7%	(-5.1%	9.9%)	-2.6%	(-56.4%	22.0%)	-8.3%	(-234.8%	198.1%)
**Anthropometric measures**									
Waist to thigh ratio	8.6%	(-0.6%	30.0%)	9.1%	(-13.2%	61.3%)	-2.9%	(-199.0%	127.9%)
BMI	0.0%	(-5.6%	5.0%)	0.3%	(-17.9%	25.2%)	0.5%	(-155.6%	150.3%)
**Clinical used blood Biomarkers**									
Total cholesterol	-0.1%	(-3.0%	2.0%)	2.8%	(-17.4%	51.7%)	2.4%	(-78.5%	155.4%)
HDL	0.0%	(-6.9%	6.0%)	-4.3%	(-37.1%	15.7%)	-0.6%	(-123.2%	153.3%)
LDL	-0.4%	(-4.3%	1.6%)	0.1%	(-36.5%	39.8%)	0.2%	(-85.9%	107.6%)
Glucose	-0.2%	(-9.6%	5.6%)	-4.8%	(-55.3%	65.6%)	3.3%	(-180.6%	288.1%)
C-reactive protein	2.2%	(-5.3%	19.4%)	-14.3%	(-189.0%	103.1%)	1.2%	(-88.4%	92.8%)

Supplementary Table 24. Average mediated proportions for DNA methylation and clinical mediators on the relationship between education attainment and all-cause mortality among White participants of NHANES 1999-2002 (n=943). Bold indicates statistical significance (p-value<0.05), and bold with two asterisks indicates significance passed Bonferroni adjustment (p-value<0.0021).

	Model 1. Less than high school (N=193) vs. College degree or higher (N=237)			Model 2. High school graduate/GED (N=285) vs. College degree or higher (N=237)			Model 3. Some college or associates degree (N=228) vs. College degree or higher (N=237)		
	Average mediated proption	(95% CI)		Average mediated proption	(95% CI)		Average mediated proption	(95% CI)	
**Chronological age clocks**									
Hannum	1.6%	(-0.6%	5.8%)	4.2%	(-0.2%	14.7%)	-0.3%	(-8.3%	6.4%)
Hovarth	-0.1%	(-2.3%	1.7%)	-1.3%	(-8.9%	1.6%)	**-3.2%**	**(-10.6% -**	**0.1%)**
Weidner	-0.2%	(-4.3%	1.9%)	0.1%	(-3.2%	3.9%)	-0.4%	(-5.0%	1.8%)
Lin	0.0%	(-3.0%	2.3%)	-1.5%	(-6.8%	1.2%)	0.0%	(-5.5%	4.4%)
Vidal-Bralo	-0.2%	(-3.6%	2.3%)	-0.2%	(-5.1%	2.6%)	-0.2%	(-4.5%	2.9%)
SkinBlood	0.5%	(-1.6%	4.2%)	0.7%	(-2.1%	5.9%)	0.0%	(-4.0%	3.3%)
Zhang	0.8%	(-1.2%	4.7%)	0.2%	(-1.8%	3.5%)	0.1%	(-3.7%	4.1%)
**Physiological functioning clocks**									
PhenoAge	**6.8%**	**(1.3%**	**16.1%)**	2.7%	(-2.1%	12.5%)	1.4%	(-5.2%	9.6%)
GrimAge	**26.0%****	**(12.9%**	**43.8%)**	**16.3%****	**(5.4%**	**40.2%)**	**31.5%****	**(16.9%**	**56.4%)**
GrimAge2	**28.7%****	**(13.9%**	**47.8%)**	**18.1%****	**(7.0%**	**45.9%)**	**30.5%****	**(16.9%**	**55.8%)**
**Pace of aging clock**									
DunedinPoAm	**13.2%****	**(4.7%**	**26.2%)**	**10.0%**	**(1.2%**	**26.2%)**	**19.8%****	**(9.0%**	**35.1%)**
**Clocks trained on other aging biomarkers**									
Yang	-1.7%	(-6.8%	2.2%)	0.5%	(-4.4%	6.4%)	0.4%	(-1.8%	5.1%)
Telomere	**10.7%**	**(3.7%**	**20.5%)**	5.5%	**(1.1%**	**13.7%)**	3.3%	(-1.0%	9.5%)
**Behavioral/Lifestyle measures**									
Sedentary behavior	**11.5%**	**(3.0%**	**27.1%)**	**19.0%****	**(7.7%**	**39.7%)**	3.1%	(-4.8%	12.7%)
Healthy eating index	-1.2%	(-14.7%	20.4%)	-1.5%	(-21.9%	11.3%)	2.2%	(-7.9%	11.4%)
Smoking pack years	**15.9%****	**(6.0%**	**30.4%)**	**11.1%****	**(3.1%**	**29.6%)**	**16.6%****	**(6.9%**	**32.7%)**
Alcohol drinker	3.0%	(-3.5%	12.0%)	6.2%	(-0.3%	19.3%)	1.9%	(-4.4%	10.7%)
**Anthropometric measures**									
Waist to thigh ratio	**7.9%**	**(1.1%**	**18.8%)**	3.1%	(-4.5%	13.8%)	4.0%	(-1.8%	14.2%)
BMI	-0.9%	(-11.4%	8.6%)	2.8%	(-3.3%	13.4%)	2.8%	(-9.2%	18.9%)
**Clinical used blood Biomarkers**									
Total cholesterol	0.5%	(-2.1%	5.0%)	-0.8%	(-20.2%	9.1%)	-2.9%	(-16.9%	4.8%)
HDL	-0.1%	(-9.5%	8.3%)	0.4%	(-4.6%	6.9%)	-0.1%	(-4.5%	3.1%)
LDL	0.6%	(-2.2%	5.0%)	-0.4%	(-17.8%	12.3%)	-4.2%	(-15.6%	2.0%)
Glucose	1.4%	(-2.7%	8.4%)	0.9%	(-2.1%	7.1%)	-1.8%	(-10.7%	2.2%)
C-reactive protein	2.7%	(-1.0%	7.6%)	0.6%	(-4.0%	5.7%)	0.8%	(-3.6%	6.5%)

Supplementary Table 25. Average mediated proportions for DNA methylation and clinical mediators on the relationship between education attainment and all-cause mortality among Hispanic participants of NHANES 1999-2002 (n=860). Bold indicates statistical significance (p-value<0.05), and bold with two asterisks indicates significance passed Bonferroni adjustment (p-value<0.0021).

	**Model 1. Less than high school (N=617) vs. College degree or higher (N=48)**		**Model 2. High school graduate/GED (N=86) vs. College degree or higher (N=48)**		**Model 3. Some college or associates degree (N=109) vs. College degree or higher (N=48)**	
	**Average mediated proption**	**(95% CI)**	**Average mediated proption**	**(95% CI)**	**Average mediated proption**	**(95% CI)**
**Chronological age clocks**						
Hannum	0.0%	(-19.5% 18.0%)	0.6%	(-16.0% 26.9%)	-0.5%	(-24.6% 26.8%)
Hovarth	0.3%	(-54.0% 40.7%)	12.5%	(-69.7% 77.1%)	9.3%	(-32.4% 55.0%)
Weidner	0.9%	(-14.9% 18.4%)	7.3%	(-28.3% 52.2%)	9.9%	(-50.2% 96.4%)
Lin	1.1%	(-42.7% 88.1%)	4.9%	(-23.5% 67.8%)	2.3%	(-20.4% 30.2%)
Vidal-Bralo	2.0%	(-42.0% 39.3%)	4.5%	(-41.0% 57.2%)	6.1%	(-9.4% 53.6%)
SkinBlood	-0.6%	(-42.8% 55.1%)	1.0%	(-19.3% 14.5%)	-0.5%	(-31.9% 20.1%)
Zhang	0.3%	(-31.1% 33.8%)	5.6%	(-27.5% 46.0%)	0.2%	(-51.4% 49.0%)
**Physiological functioning clocks**						
PhenoAge	1.5%	(-32.4% 34.4%)	4.5%	(-14.2% 30.5%)	1.3%	(-19.5% 19.1%)
GrimAge	1.1%	(-47.7% 56.3%)	0.1%	(-24.2% 29.9%)	-1.7%	(-130.7% 138.5%)
GrimAge2	0.9%	(-60.5% 69.9%)	0.1%	(-21.7% 23.8%)	-1.7%	(-107.2% 79.9%)
**Pace of aging clock**						
DunedinPoAm	-0.1%	(-31.6% 29.2%)	-0.6%	(-41.2% 35.8%)	-3.3%	(-176.9% 125.4%)
**Clocks trained on other aging biomarkers**						
Yang	0.0%	(-17.0% 19.8%)	2.4%	(-17.8% 44.5%)	8.0%	(-24.0% 41.1%)
Telomere	1.9%	(-21.2% 30.0%)	5.2%	(-58.3% 60.9%)	8.8%	(-7.1% 41.1%)
**Behavioral/Lifestyle measures**						
Sedentary behavior	-8.2%	(-124.0% 123.7%)	-4.0%	(-70.7% 21.9%)	-11.2%	(-99.9% 9.9%)
Healthy eating index	-4.7%	(-65.3% 80.7%)	-7.4%	(-80.6% 66.8%)	-2.7%	(-31.1% 18.4%)
Smoking pack years	0.1%	(-28.7% 23.7%)	-0.8%	(-42.9% 26.2%)	0.2%	(-13.9% 38.2%)
Alcohol drinker	0.0%	(-115.8% 111.1%)	-4.3%	(-99.3% 77.4%)	-1.1%	(-33.5% 23.5%)
**Anthropometric measures**						
Waist to thigh ratio	-4.7%	(-69.7% 59.2%)	0.9%	(-31.9% 44.7%)	1.1%	(-14.1% 22.4%)
BMI	1.4%	(-43.7% 69.7%)	0.6%	(-58.4% 61.1%)	-1.8%	(-41.4% 35.4%)
**Clinical used blood Biomarkers**						
Total cholesterol	-0.4%	(-59.7% 23.4%)	2.3%	(-63.3% 95.1%)	0.4%	(-81.8% 90.4%)
HDL	-3.2%	(-62.7% 45.2%)	-0.1%	(-28.4% 14.7%)	-0.8%	(-36.5% 17.3%)
LDL	-0.7%	(-42.8% 43.6%)	3.0%	(-49.7% 78.0%)	0.2%	(-81.1% 101.3%)
Glucose	-4.3%	(-119.3% 70.3%)	-1.2%	(-120.6%114.7%)	-2.4%	(-36.1% 27.1%)
C-reactive protein	0.1%	(-5.7% 8.1%)	0.4%	(-17.6% 15.7%)	0.6%	(-3.5% 8.3%)

Supplementary Table 26. Average mediated proportions for DNA methylation and clinical mediators on the relationship between household income and all-cause mortality among Black participants of NHANES 1999-2002 (n=516). Bold indicates statistical significance (p-value<0.05), and bold with two asterisks indicates significance passed Bonferroni adjustment (p-value<0.0021).

	Model 1. PIR<1 (N=86) vs. PIR 5+ (N=54)		Model 2. PIR 1-2 (N=155) vs. PIR 5+ (N=54)		Model 3. PIR 2-5 (N=221) vs. PIR 5+ (N=54)	
	Average mediated proption	(95% CI)	Average mediated proption	(95% CI)	Average mediated proption	(95% CI)
**Chronological age clocks**						
Hannum	2.9%	(-8.0% 17.8%)	8.0%	(-8.5% 28.4%)	-4.5%	(-89.1% 34.9%)
Hovarth	-2.4%	(-26.7% 7.7%)	3.9%	(-7.2% 30.9%)	-0.6%	(-105.6% 44.2%)
Weidner	0.2%	(-2.9% 7.8%)	0.1%	(-5.2% 7.0%)	1.0%	(-17.2% 26.6%)
Lin	-0.5%	(-13.8% 6.2%)	-0.9%	(-17.7% 10.9%)	-0.5%	(-32.9% 22.3%)
Vidal-Bralo	0.6%	(-2.9% 8.2%)	-1.4%	(-15.5% 5.4%)	-0.5%	(-12.0% 3.2%)
SkinBlood	0.0%	(-7.4% 6.8%)	2.4%	(-3.7% 17.7%)	1.6%	(-34.0% 26.7%)
Zhang	0.7%	(-10.4% 13.7%)	4.4%	(-5.2% 26.3%)	1.0%	(-27.0% 31.1%)
**Physiological functioning clocks**						
PhenoAge	1.1%	(-3.9% 13.3%)	6.1%	(-6.6% 26.4%)	-4.5%	(-90.5% 19.4%)
GrimAge	**17.6%****	**(6.7% 43.2%)**	**23.5%**	**(8.0% 75.6%)**	7.2%	(-15.9% 42.8%)
GrimAge2	**20.6%****	**(8.7% 42.1%)**	**28.8%**	**(13.4% 78.5%)**	7.1%	(-18.7% 52.8%)
**Pace of aging clock**						
DunedinPoAm	0.0%	(-5.4% 4.9%)	7.7%	(-5.5% 25.7%)	-2.1%	(-36.1% 18.6%)
**Clocks trained on other aging biomarkers**						
Yang	0.9%	(-3.7% 8.6%)	0.5%	(-4.0% 9.0%)	0.2%	(-8.6% 12.5%)
Telomere	**10.8%**	**(2.7% 26.0%)**	**9.2%**	**(0.9% 29.5%)**	6.2%	(-20.5% 47.0%)
**Behavioral/Lifestyle measures**						
Sedentary behavior	-3.7%	(-24.8% 12.9%)	4.1%	(-7.9% 30.7%)	10.0%	(-22.4% 69.4%)
Healthy eating index	-1.0%	(-9.6% 6.0%)	-0.7%	(-8.0% 2.1%)	-7.6%	(-58.1% 14.2%)
Smoking pack years	**3.1%**	**(0.4% 8.4%)**	1.4%	(-2.5% 10.6%)	3.3%	(-5.3% 23.3%)
Alcohol drinker	-1.8%	(-13.7% 13.4%)	0.8%	(-5.7% 12.8%)	0.0%	(-24.8% 23.1%)
**Anthropometric measures**						
Waist to thigh ratio	6.1%	(-0.5% 23.8%)	**12.6%**	**(1.0% 57.5%)**	0.1%	(-18.3% 12.2%)
BMI	-1.6%	(-30.3% 7.1%)	0.1%	(-13.0% 12.0%)	-0.1%	(-18.9% 14.1%)
**Clinical used blood Biomarkers**						
Total cholesterol	1.4%	(-12.6% 16.7%)	-0.7%	(-9.9% 4.4%)	-0.3%	(-21.0% 13.3%)
HDL	-1.8%	(-19.1% 4.5%)	-4.3%	(-47.8% 6.7%)	0.0%	(-16.0% 14.1%)
LDL	1.3%	(-8.3% 17.3%)	-1.8%	(-15.5% 5.0%)	-1.9%	(-37.1% 6.9%)
Glucose	1.3%	(-5.8% 17.2%)	0.2%	(-5.7% 6.4%)	-0.4%	(-16.6% 11.7%)
C-reactive protein	-1.5%	(-13.2% 9.2%)	-0.3%	(-10.4% 10.3%)	-0.2%	(-13.0% 8.9%)

Supplementary Table 27. Average mediated proportions for DNA methylation and clinical mediators on the relationship between household income and all-cause mortality among White participants of NHANES 1999-2002 (n=943). Bold indicates statistical significance (p-value<0.05), and bold with two asterisks indicates significance passed Bonferroni adjustment (p-value<0.0021).

	Model 1. PIR<1 (N=69) vs. PIR 5+ (N=261)		Model 2. PIR 1-2 (N=184) vs. PIR 5+ (N=261)		Model 3. PIR 2-5 (N=429) vs. PIR 5+ (N= 261)	
	Average mediated proption	(95% CI)	Average mediated proption	(95% CI)	Average mediated proption	(95% CI)
**Chronological age clocks**						
Hannum	4.4%	(-0.9% 15.9%)	3.5%	(-0.4% 9.4%)	0.2%	(-8.8% 9.8%)
Hovarth	-0.2%	(-5.9% 5.7%)	1.0%	(-3.1% 5.5%)	-0.6%	(-8.5% 4.2%)
Weidner	-0.1%	(-3.9% 2.9%)	1.8%	(-0.9% 5.7%)	0.0%	(-2.8% 2.3%)
Lin	0.1%	(-3.9% 4.8%)	-0.5%	(-2.9% 0.9%)	-1.4%	(-12.6% 2.6%)
Vidal-Bralo	0.4%	(-2.5% 7.1%)	2.2%	(-0.6% 7.1%)	0.2%	(-2.9% 4.4%)
SkinBlood	0.0%	(-6.0% 4.6%)	0.0%	(-2.3% 2.3%)	-1.7%	(-9.1% 2.8%)
Zhang	0.0%	(-3.6% 3.9%)	-0.2%	(-2.1% 1.2%)	-1.9%	(-10.2% 2.1%)
**Physiological functioning clocks**						
PhenoAge	**8.7%**	**(0.2% 29.6%)**	**9.2%**	**(1.8% 21.3%)**	4.8%	(-1.4% 19.4%)
GrimAge	**38.5%****	**(19.1% 64.4%)**	**28.2%****	**(14.4% 55.6%)**	**25.9%**	**(6.2% 79.0%)**
GrimAge2	**41.7%****	**(21.8% 71.1%)**	**31.0%****	**(16.8% 60.1%)**	**28.4%****	**(10.5% 95.1%)**
**Pace of aging clock**						
DunedinPoAm	**25.2%****	**(6.4% 53.4%)**	**15.2%****	**(4.1% 38.8%)**	**16.7%**	**(4.1% 58.6%)**
**Clocks trained on other aging biomarkers**						
Yang	0.6%	(-4.0% 8.5%)	0.8%	(-1.5% 5.3%)	0.0%	(-4.0% 5.1%)
Telomere	3.9%	(-4.5% 14.5%)	**8.9%****	**(2.9% 20.8%)**	**6.1%**	**(0.8% 20.4%)**
**Behavioral/Lifestyle measures**						
Sedentary behavior	**29.0%**	**(6.1% 63.3%)**	**12.8%**	**(2.8% 41.8%)**	12.0%	(-0.8% 56.8%)
Healthy eating index	1.9%	(-21.4% 25.3%)	3.9%	(-3.2% 18.3%)	1.2%	(-7.8% 20.5%)
Smoking pack years	**15.4%****	**(5.3% 30.5%)**	**17.1%****	**(6.4% 42.4%)**	**17.1%****	**(6.0% 55.7%)**
Alcohol drinker	-8.0%	(-33.1% 8.5%)	3.0%	(-14.5% 18.8%)	7.3%	(-0.6% 24.8%)
**Anthropometric measures**						
Waist to thigh ratio	2.6%	(-15.5% 15.2%)	4.7%	(-7.1% 17.5%)	**12.4%**	**(1.9% 35.2%)**
BMI	-1.0%	(-14.9% 13.2%)	-2.0%	(-19.9% 6.5%)	1.1%	(-19.7% 20.3%)
**Clinical used blood Biomarkers**						
Total cholesterol	0.2%	(-6.4% 8.3%)	1.4%	(-1.9% 9.8%)	0.0%	(-6.0% 8.7%)
HDL	-5.8%	(-21.7% 6.4%)	1.2%	(-8.0% 11.2%)	1.4%	(-8.3% 14.2%)
LDL	-2.2%	(-16.3% 6.2%)	1.2%	(-2.6% 8.9%)	-0.6%	(-10.3% 11.2%)
Glucose	4.7%	(-0.4% 14.3%)	0.3%	(-3.6% 6.6%)	4.8%	(-1.9% 20.1%)
C-reactive protein	**9.0%**	**(1.1% 21.7%)**	**6.6%**	**(1.1% 15.6%)**	2.3%	(-0.9% 13.3%)

Supplementary Table 28. Average mediated proportions for DNA methylation and clinical mediators on the relationship between household income and all-cause mortality among Hispanic participants of NHANES 1999-2002 (n=860). Bold indicates statistical significance (p-value<0.05), and bold with two asterisks indicates significance passed Bonferroni adjustment (p-value<0.0021).

	Model 1. PIR<1 (N=198) vs. PIR 5+ (N=66)		Model 2. PIR 1-2 (N=355) vs. PIR 5+ (N=66)		Model 3. PIR 2-5 (N=241) vs. PIR 5+ (N=66)	
	Average mediated proption	(95% CI)	Average mediated proption	(95% CI)	Average mediated proption	(95% CI)
**Chronological age clocks**						
Hannum	0.3%	(-104.5%130.4%)	0.0%	(-38.9% 27.4%)	0.5%	(-51.5% 54.5%)
Hovarth	2.9%	(-76.2% 107.5%)	0.1%	(-60.9% 87.3%)	0.4%	(-28.7% 51.4%)
Weidner	2.9%	(-36.8% 54.4%)	0.3%	(-128.7% 66.8%)	3.5%	(-66.8% 90.7%)
Lin	2.2%	(-303.7%231.7%)	-1.0%	(-55.3% 50.8%)	1.0%	(-46.4% 41.8%)
Vidal-Bralo	10.0%	(-152.6%170.5%)	-9.1%	(-236.8%184.4%)	4.1%	(-151.3%124.7%)
SkinBlood	0.2%	(-55.8% 52.4%)	-0.1%	(-28.7% 19.8%)	0.8%	(-108.2% 56.8%)
Zhang	-0.3%	(-133.3%127.1%)	0.4%	(-34.2% 31.3%)	0.5%	(-40.8% 54.3%)
**Physiological functioning clocks**						
PhenoAge	1.0%	(-42.5% 66.1%)	3.8%	(-105.2% 86.9%)	0.3%	(-60.3% 64.5%)
GrimAge	2.5%	(-35.5% 53.1%)	2.2%	(-78.1% 87.2%)	0.2%	(-65.1% 66.0%)
GrimAge2	1.0%	(-44.5% 54.9%)	4.6%	(-163.5%163.6%)	-0.1%	(-73.7% 55.7%)
**Pace of aging clock**						
DunedinPoAm	-3.7%	(-184.1%130.8%)	0.4%	(-70.0% 77.1%)	0.1%	(-55.4% 68.9%)
**Clocks trained on other aging biomarkers**						
Yang	0.0%	(-86.4% 55.6%)	2.9%	(-48.0% 51.3%)	0.7%	(-48.2% 65.4%)
Telomere	2.4%	(-99.4% 96.9%)	1.2%	(-112.0% 67.8%)	-0.1%	(-53.0% 49.8%)
**Behavioral/Lifestyle measures**						
Sedentary behavior	0.7%	(-408.7%392.8%)	14.6%	(-242.4%240.5%)	-1.5%	(-223.0%226.0%)
Healthy eating index	10.7%	(-524.5%707.7%)	-3.1%	(-95.9% 66.3%)	-2.9%	(-87.6% 75.1%)
Smoking pack years	6.0%	(-195.8%186.7%)	0.3%	(-26.6% 34.0%)	0.0%	(-30.2% 39.9%)
Alcohol drinker	-2.3%	(-283.6%221.7%)	0.0%	(-157.1%143.2%)	1.8%	(-46.7% 46.9%)
**Anthropometric measures**						
Waist to thigh ratio	4.7%	(-145.0%201.2%)	10.5%	(-147.4%319.6%)	-1.0%	(-63.7% 84.8%)
BMI	1.7%	(-86.4% 74.3%)	6.3%	(-121.9%110.8%)	-1.0%	(-150.3% 87.5%)
**Clinical used blood Biomarkers**						
Total cholesterol	-0.1%	(-165.2%133.3%)	2.7%	(-52.4% 65.4%)	50.8%	(-578.4%814.2%)
HDL	2.5%	(-85.6% 76.3%)	0.7%	(-84.6% 59.9%)	0.1%	(-37.4% 65.9%)
LDL	-0.4%	(-151.6%127.8%)	2.6%	(-89.9% 63.6%)	47.5%	(-769.7%824.3%)
Glucose	0.6%	(-144.3%134.7%)	0.7%	(-54.9% 73.8%)	0.2%	(-71.9% 59.9%)
C-reactive protein	0.0%	(-31.4% 35.8%)	-1.7%	(-105.4% 70.2%)	-1.4%	(-50.6% 46.4%)
