# Supplementary material for: The mediating role of DNA methylation clocks in associations of race, ethnicity, education, income, and occupation with mortality: findings from NHANES 1999-2002
Source: Aging (Albany NY). 2026 May 8;18(1):466–512. doi: 10.18632/aging.206377 (PMC13632862; doi:10.18632/aging.206377)
Supplement: Supplementary file 7 [file aging-18-1-206377-s017.pdf]

Supplementary Table 32. Average mediated proportions for ten components of GrimAge2 on the relationship between race and ethnicity and all-cause mortality, NHANES 1999-2002 (n=2,402). Bold indicates statistical significance (p-value<0.05), and bold with two asterisks indicates significance passed Bonferroni adjustment (p-value<0.0021).

	Model 1. Black (N= 516) vs. White (N= 943)		Model 2. Hispanic (N=860) vs. White (N= 943)		Model 3. Other races (N=83) vs. White (N= 943)	
	Average mediated proportion	(95% CI)	Average mediated proportion	(95% CI)	Average mediated proportion	(95% CI)
**GrimAge2 components**						
GDF15Mort	**4.1%**	**(0.1% 11.7%)**	**16.3%**	**(4.2% 56.1%)**	8.1%	(-126.9% 131.2%)
B2MMort	**25.3%****	**(11.5% 66.4%)**	**22.2%****	**(9.0% 70.8%)**	32.2%	(-479.1% 568.0%)
CystatinCMort	**12.1%****	**(5.1% 27.8%)**	**27.3%**	**(10.7% 84.0%)**	54.4%	(-625.8% 1107.9%)
TIMP1Mort	8.6%	(-1.1% 27.2%)	**13.2%**	**(0.9% 43.6%)**	6.9%	(-124.1% 186.7%)
ADMMort	**-21.4%****	**(-52.2% -9.1%)**	-13.8%	(-64.6% 0.3%)	-2.8%	(-72.8% 57.9%)
PAI1Mort	-1.4%	(-8.9% 3.4%)	0.4%	(-10.3% 10.1%)	-1.6%	(-64.9% 55.8%)
LeptinMort	**5.6%**	**(0.6% 15.9%)**	2.1%	(-3.0% 12.0%)	1.0%	(-52.9% 44.4%)
PACKYRSMort	**38.7%****	**(22.0% 75.4%)**	0.4%	(-58.0% 25.5%)	-5.9%	(-182.8% 247.6%)
CRPMort	**20.5%****	**(9.1% 45.9%)**	**30.8%****	**(13.6% 80.6%)**	11.7%	(-126.0% 137.7%)
logA1CMort	**27.7%****	**(14.5% 52.2%)**	**29.5%****	**(12.4% 121.6%)**	21.9%	(-184.9% 234.8%)

Supplementary Table 33. Average mediated proportions for ten components of GrimAge2 on the relationship between education attainment and all-cause mortality, NHANES 1999-2002 (n=2,402). Bold indicates statistical significance (p-value<0.05), and bold with two asterisks indicates significance passed Bonferroni adjustment (p-value<0.0021).

	Model 1. Less than high school (N=1,096) vs. College degree or higher (N= 370)		Model 2. High school graduate/GED (N=496) vs. College degree or higher (N= 370)		Model 3. Some college or associates degree (N=440) vs. College degree or higher (N= 370)	
	Average mediated proportion	(95% CI)	Average mediated proportion	(95% CI)	Average mediated proportion	(95% CI)
**GrimAge2 components**						
GDF15Mort	**7.0%****	**(2.2% 13.5%)**	3.1%	(-1.2% 11.8%)	**10.8%****	**(4.3% 21.4%)**
B2MMort	**4.0%**	**(1.1% 8.0%)**	**5.4%**	**(0.6% 13.6%)**	2.6%	(-4.3% 10.6%)
CystatinCMort	**4.1%**	**(0.7% 9.8%)**	3.3%	(-0.9% 12.7%)	4.2%	(-2.6% 12.8%)
TIMP1Mort	**8.7%****	**(3.4% 16.5%)**	3.4%	(-0.2% 13.4%)	**9.5%**	**(2.4% 19.7%)**
ADMMort	1.4%	(-2.8% 6.2%)	-0.9%	(-9.1% 4.9%)	1.8%	(-4.8% 10.0%)
PAI1Mort	**3.0%**	**(0.0% 8.5%)**	2.1%	(-0.6% 8.4%)	**8.6%**	**(1.0% 26.3%)**
LeptinMort	1.3%	(-2.8% 7.8%)	2.5%	(-2.7% 11.2%)	0.0%	(-3.9% 5.3%)
PACKYRSMort	**17.6%****	**(8.8% 30.2%)**	**17.3%****	**(6.3% 37.1%)**	**27.0%****	**(12.5% 55.8%)**
CRPMort	**14.4%****	**(6.2% 27.5%)**	**8.2%****	**(2.3% 23.6%)**	**9.4%****	**(2.3% 22.6%)**
logA1CMort	5.7%	(-1.0% 14.9%)	**7.0%**	**(0.7% 21.3%)**	**7.4%**	**(0.2% 24.2%)**

Supplementary Table 34. Average mediated proportions for ten components of GrimAge2 on the relationship between household income and all-cause mortality, NHANES 1999-2002 (n=2,402). Bold indicates statistical significance (p-value<0.05), and bold with two asterisks indicates significance passed Bonferroni adjustment (p-value<0.0021).

	Model 1. PIR<1 (N=373) vs. PIR 5+ (N= 399)		Model 2. PIR 1-2 (N=707) vs. PIR 5+ (N= 399)		Model 3. PIR 2-5 (N=923) vs. PIR 5+ (N= 399)	
	Average mediated proportion	(95% CI)	Average mediated proportion	(95% CI)	Average mediated proportion	(95% CI)
**GrimAge2 components**						
GDF15Mort	4.5%	(-1.7% 13.2%)	**5.9%****	**(2.2% 13.2%)**	**4.5%**	**(0.5% 18.4%)**
B2MMort	**9.0%****	**(2.8% 18.9%)**	**8.2%****	**(3.5% 15.0%)**	**10.0%****	**(3.8% 24.9%)**
CystatinCMort	**6.9%**	**(1.3% 15.9%)**	**9.3%****	**(4.0% 19.8%)**	**5.7%**	**(1.1% 17.7%)**
TIMP1Mort	**9.4%**	**(2.4% 19.9%)**	**10.2%****	**(4.2% 20.2%)**	**6.5%**	**(0.0% 21.0%)**
ADMMort	2.1%	(-3.4% 8.6%)	**4.4%**	**(0.6% 11.3%)**	**3.5%**	**(-6.5% 14.5%)**
PAI1Mort	1.9%	(-3.2% 8.8%)	1.9%	(-0.4% 6.1%)	3.8%	(-0.5% 14.5%)
LeptinMort	0.6%	(-1.5% 4.7%)	1.5%	(-1.3% 7.0%)	-0.4%	(-4.9% 2.7%)
PACKYRSMort	**24.0%****	**(12.9% 40.2%)**	**17.4%****	**(8.7% 32.6%)**	**14.6%**	**(3.8% 38.5%)**
CRPMort	**12.9%**	**(1.8% 27.8%)**	**16.9%****	**(7.3% 32.4%)**	**10.2%**	**(1.3% 29.4%)**
logA1CMort	**8.7%**	**(0.7% 19.0%)**	**5.6%**	**(1.1% 13.6%)**	**8.4%**	**(1.9% 23.7%)**
