# Supplementary material for: The mediating role of DNA methylation clocks in associations of race, ethnicity, education, income, and occupation with mortality: findings from NHANES 1999-2002
Source: Aging (Albany NY). 2026 May 8;18(1):466–512. doi: 10.18632/aging.206377 (PMC13632862; doi:10.18632/aging.206377)
Supplement: Supplementary file 8 [file aging-18-1-206377-s019.pdf]

Supplementary Table 36. Average mediated proportions for DNA methylation and clinical mediators on the relationship between race and ethnicity and all-cause mortality, among completed cases from NHANES 1999-2002 cohort (n=2,401). Bold indicates statistical significance (p-value<0.05), and bold with two asterisks indicates significance passed Bonferroni adjustment (p-value<0.0021).

	Model 1. Black (N= 516) vs. White (N= 943)		Model 2. Hispanic (N=859) vs. White (N= 943)		Model 3. Other races (N=83) vs. White (N= 943)	
	Average mediated proportion	(95% CI)	Average mediated proportion	(95% CI)	Average mediated proportion	(95% CI)
**Chronological age clocks**						
Hannum	**-19.1%****	**(-37.8%- 10.5%)**	5.8%	(-4.4% 20.7%)	4.5%	(-100.1% 99.9%)
Hovarth	**-5.2%**	**(-14.6% - 0.7%)**	-4.9%	(-23.4% 1.3%)	-1.3%	(-93.6% 56.7%)
Weidner	**-2.5%**	**(-8.2% - 0.3%)**	-3.9%	(-17.5% 0.6%)	-4.9%	(-98.1% 82.1%)
Lin	-2.9%	(-10.6% 0.3%)	-3.5%	(-15.7% 1.7%)	-3.8%	(-62.5% 73.0%)
Vidal-Bralo	**-14.6%****	**(-35.9% - 4.9%)**	**-10.2%**	**(-40.9% - 2.4%)**	-13.5%	(-251.5%159.5%)
SkinBlood	**-4.9%**	**(-15.5% - 0.5%)**	0.1%	(-4.1% 4.4%)	1.0%	(-38.1% 47.3%)
Zhang	**-9.9%**	**(-27.0% - 2.8%)**	-1.1%	(-8.2% 2.4%)	-2.4%	(-59.4% 50.6%)
**Physiological functioning clocks**						
PhenoAge	-6.8%	(-22.8% 0.6%)	9.4%	(-1.7% 36.3%)	5.7%	(-119.3%161.6%)
GrimAge	**34.4%****	**(18.7% 66.8%)**	15.7%	(-16.5% 52.8%)	12.0%	(-193.5%234.7%)
GrimAge2	**46.0%****	**(26.6% 88.7%)**	**39.3%**	**(17.6% 120.0%)**	24.8%	(-206.7%288.7%)
**Pace of aging clock**						
DunedinPoAm	**23.6%****	**(10.0% 48.9%)**	**29.4%**	**(11.2% 83.1%)**	27.2%	(-211.4%340.7%)
**Clocks trained on other aging biomarkers**						
Yang	7.4%	(-6.8% 20.9%)	3.8%	(-9.4% 19.7%)	1.3%	(-98.4% 124.6%)
Telomere	**-44.4%****	**(-79.1%- 27.2%)**	2.5%	(-19.4% 19.3%)	9.1%	(-177.4%157.2%)
**Behavioral/Lifestyle measures**						
Sedentary behavior	**27.8%****	**(13.4% 54.0%)**	**24.1%**	**(5.9% 80.7%)**	18.3%	(-258.0%230.9%)
Healthy eating index	**6.6%**	**(0.7% 18.7%)**	3.3%	(-6.4% 24.3%)	-0.1%	(-39.6% 42.9%)
Smoking pack years	**-19.0%****	**(-45.3% - 8.5%)**	**-15.0%**	**(-48.0% - 5.1%)**	-10.4%	(-155.5%171.8%)
Alcohol drinker	**13.6%****	**(5.4% 27.6%)**	**10.7%**	**(1.5% 49.2%)**	20.6%	(-334.4%457.4%)
**Anthropometric measures**						
Waist to thigh ratio	**-33.7%****	**(-86.4%- 17.0%)**	**16.4%**	**(4.3% 59.3%)**	7.7%	(-240.8%251.2%)
BMI	2.3%	(-7.1% 15.0%)	0.3%	(-17.2% 18.0%)	-0.2%	(-28.1% 30.2%)
**Clinical used blood Biomarkers**						
Total cholesterol	-0.6%	(-4.5% 1.3%)	0.0%	(-4.7% 3.6%)	-0.3%	(-35.1% 20.0%)
HDL	-0.8%	(-6.6% 2.8%)	5.7%	(-5.0% 17.9%)	1.4%	(-40.2% 41.0%)
LDL	0.0%	(-4.6% 5.1%)	0.1%	(-6.0% 5.7%)	0.1%	(-6.6% 15.0%)
Glucose	**7.7%**	**(1.9% 17.9%)**	**5.6%**	**(1.0% 19.6%)**	7.1%	(-137.9% 92.1%)
C-reactive protein	**9.5%****	**(3.0% 23.0%)**	1.7%	(-2.2% 8.8%)	-0.7%	(-56.2% 57.8%)

Supplementary Table 37. Average mediated proportions for DNA methylation and clinical mediators on the relationship between education attainment and all-cause mortality, among completed cases from NHANES 1999-2002 cohort (n=2,399). Bold indicates statistical significance (p-value<0.05), and bold with two asterisks indicates significance passed Bonferroni adjustment (p-value<0.0021).

	Model 1. Less than high school (N=1,093) vs. College degree or higher (N= 370)			Model 2. High school graduate/GED (N=496) vs. College degree or higher (N= 370)			Model 3. Some college or associates degree (N=440) vs. College degree or higher (N= 370)		
	Average mediated proption	(95% CI)		Average mediated proption	(95% CI)		Average mediated proption	(95% CI)	
**Chronological age clocks**									
Hannum	1.7%	(-0.1%	4.4%)	**4.4%**	**(0.3%**	**13.4%)**	0.5%	(-6.9%	7.7%)
Hovarth	-0.1%	(-1.9%	1.3%)	-1.7%	(-8.9%	1.3%)	**-4.1%**	**(-14.0%**	**- 0.2%)**
Weidner	-0.2%	(-3.2%	1.1%)	0.1%	(-3.0%	3.7%)	-0.9%	(-7.8%	1.6%)
Lin	-0.2%	(-2.2%	1.2%)	-1.2%	(-5.5%	1.0%)	-0.5%	(-5.4%	3.1%)
Vidal-Bralo	-0.7%	(-3.3%	0.6%)	-0.3%	(-5.0%	1.6%)	-0.7%	(-5.8%	2.6%)
SkinBlood	0.3%	(-0.9%	2.4%)	0.9%	(-1.2%	5.7%)	0.0%	(-3.4%	2.7%)
Zhang	0.5%	(-0.8%	2.4%)	0.2%	(-1.7%	3.1%)	-0.1%	(-5.5%	3.2%)
**Physiological functioning clocks**									
PhenoAge	**6.8%****	**(2.8%**	**13.4%)**	2.9%	(-1.3%	12.2%)	3.1%	(-5.0%	12.4%)
GrimAge	**23.5%****	**(13.2%**	**37.0%)**	**18.7%****	**(8.1%**	**41.7%)**	**31.8%****	**(17.8%**	**57.5%)**
GrimAge2	**28.1%****	**(16.9%**	**42.9%)**	**21.4%****	**(9.9%**	**48.5%)**	**32.4%****	**(17.2%**	**64.2%)**
**Pace of aging clock**									
DunedinPoAm	**13.3%****	**(6.1%**	**22.5%)**	**11.4%****	**(2.8%**	**27.6%)**	**22.0%****	**(11.0%**	**38.0%)**
**Clocks trained on other aging biomarkers**									
Yang	-0.8%	(-4.2%	2.3%)	1.2%	(-1.5%	6.0%)	0.3%	(-3.9%	4.5%)
Telomere	**5.3%****	**(1.9%**	**10.1%)**	**5.7%****	**(1.9%**	**13.1%)**	3.5%	(-0.9%	10.9%)
**Behavioral/Lifestyle measures**									
Sedentary behavior	**13.3%****	**(6.9%**	**23.5%)**	**20.8%****	**(9.8%**	**42.7%)**	5.9%	(-1.4%	19.3%)
Healthy eating index	2.3%	(-7.1%	14.1%)	1.2%	(-14.7%	13.4%)	3.8%	(-5.7%	14.4%)
Smoking pack years	**10.2%****	**(4.5%**	**18.2%)**	**11.0%**	**(2.1%**	**31.2%)**	**16.4%****	**(7.4%**	**31.0%)**
Alcohol drinker	2.6%	(-2.5%	8.5%)	**7.1%**	**(0.7%**	**19.6%)**	2.1%	(-2.5%	10.5%)
**Anthropometric measures**									
Waist to thigh ratio	**5.6%****	**(1.7%**	**12.8%)**	2.8%	(-4.7%	10.8%)	4.6%	(-2.8%	14.6%)
BMI	0.4%	(-4.6%	6.1%)	3.1%	(-2.0%	15.3%)	5.3%	(-7.0%	23.6%)
**Clinical used blood Biomarkers**									
Total cholesterol	0.0%	(-1.5%	1.7%)	-0.1%	(-13.2%	10.1%)	-3.1%	(-14.8%	2.2%)
HDL	-0.1%	(-6.6%	5.5%)	0.1%	(-4.7%	5.2%)	0.0%	(-4.4%	3.8%)
LDL	-3.8%	(-14.6%	0.5%)	10.6%	(-27.0%	60.9%)	-4.7%	(-60.0%	19.9%)
Glucose	2.4%	(0.1%	6.5%)	1.3%	(-2.0%	7.0%)	-1.7%	(-11.6%	4.1%)
C-reactive protein	**2.0%**	**(0.5%**	**4.9%)**	0.7%	(-2.3%	4.1%)	0.8%	(-3.2%	6.2%)

Supplementary Table 38. Average mediated proportions for DNA methylation and clinical mediators on the relationship between household income and all-cause mortality, among completed cases from NHANES 1999-2002 cohort (n=2,123). Bold indicates statistical significance (p-value<0.05), and bold with two asterisks indicates significance passed Bonferroni adjustment (p-value<0.0021).

	Model 1. PIR<1 (N=373) vs. PIR 5+ (N= 399)		Model 2. PIR 1-2 (N=591) vs. PIR 5+ (N= 399)		Model 3. PIR 2-5 (N=760) vs. PIR 5+ (N= 399)	
	Average mediated proption	(95% CI)	Average mediated proption	(95% CI)	Average mediated proption	(95% CI)
**Chronological age clocks**						
Hannum	3.1%	(-0.6% 7.8%)	3.8%	(-0.0% 8.2%)	-0.1%	(-7.3% 6.5%)
Hovarth	-0.1%	(-2.7% 1.4%)	-0.2%	(-4.1% 3.3%)	0.1%	(-3.8% 4.1%)
Weidner	0.3%	(-1.6% 2.9%)	1.2%	(-1.2% 4.6%)	0.0%	(-1.8% 2.5%)
Lin	0.1%	(-1.5% 2.5%)	-0.7%	(-2.9% 0.9%)	-0.9%	(-6.9% 2.7%)
Vidal-Bralo	-0.3%	(-3.0% 1.0%)	1.2%	(-1.9% 4.9%)	0.0%	(-2.3% 2.7%)
SkinBlood	-0.3%	(-3.8% 1.8%)	0.0%	(-1.7% 2.1%)	-0.2%	(-3.4% 4.3%)
Zhang	-0.4%	(-2.8% 1.2%)	-0.5%	(-2.9% 1.4%)	-0.8%	(-5.7% 1.6%)
**Physiological functioning clocks**						
PhenoAge	**5.3%**	**(0.4% 14.4%)**	**10.0%****	**(3.3% 20.3%)**	3.2%	(-1.6% 13.5%)
GrimAge	**32.6%****	**(20.6% 49.6%)**	**24.2%****	**(13.7% 42.9%)**	**19.7%**	**(7.2% 52.3%)**
GrimAge2	**37.1%****	**(22.7% 57.4%)**	**28.4%****	**(17.2% 54.3%)**	**22.7%****	**(9.3% 61.1%)**
**Pace of aging clock**						
DunedinPoAm	**19.4%****	**(9.2% 35.6%)**	**14.1%****	**(5.7% 30.3%)**	**13.3%**	**(2.0% 38.0%)**
**Clocks trained on other aging biomarkers**						
Yang	0.2%	(-4.9% 5.1%)	0.8%	(-3.2% 5.6%)	0.0%	(-3.1% 2.2%)
Telomere	2.2%	(-1.0% 7.4%)	**6.8%****	**(2.7% 13.3%)**	2.9%	(-0.7% 9.6%)
**Behavioral/Lifestyle measures**						
Sedentary behavior	**20.4%**	**(4.7% 40.8%)**	**10.9%**	**(1.9% 26.2%)**	**13.7%**	**(1.2% 46.1%)**
Healthy eating index	4.9%	(-9.6% 20.5%)	-0.2%	(-7.1% 8.6%)	4.8%	(-2.5% 26.2%)
Smoking pack years	**11.2%****	**(4.8% 19.2%)**	**11.6%**	**(3.4% 28.6%)**	**13.2%****	**(4.2% 35.4%)**
Alcohol drinker	-3.6%	(-19.8% 7.8%)	1.8%	(-11.2% 13.2%)	6.0%	(-1.6% 20.2%)
**Anthropometric measures**						
Waist to thigh ratio	4.9%	(-2.1% 12.4%)	6.1%	(-1.8% 16.3%)	**10.2%**	**(1.2% 26.3%)**
BMI	-1.1%	(-11.8% 8.3%)	0.6%	(-12.9% 8.2%)	1.6%	(-11.9% 14.3%)
**Clinical used blood Biomarkers**						
Total cholesterol	-0.1%	(-3.8% 3.4%)	0.2%	(-1.8% 3.6%)	-0.6%	(-7.1% 3.4%)
HDL	-3.8%	(-16.0% 5.4%)	0.2%	(-7.6% 7.1%)	0.7%	(-6.2% 9.4%)
LDL	-1.7%	(-15.7% 4.6%)	-0.4%	(-37.6% 25.1%)	1.4%	(-73.5% 95.2%)
Glucose	**6.2%**	**(1.3% 15.3%)**	1.7%	(-2.5% 7.6%)	3.7%	(-0.5% 14.0%)
C-reactive protein	**2.0%**	**(0.4% 5.2%)**	**4.8%**	**(0.6% 10.5%)**	2.4%	(-0.2% 7.8%)
