# Supplementary material for: The mediating role of DNA methylation clocks in associations of race, ethnicity, education, income, and occupation with mortality: findings from NHANES 1999-2002
Source: Aging (Albany NY). 2026 May 8;18(1):466–512. doi: 10.18632/aging.206377 (PMC13632862; doi:10.18632/aging.206377)
Supplement: Supplementary file 9 [file aging-18-1-206377-s022.pdf]

Supplementary Table 41. The wald-test for a square term of DNA methylation and clinical mediators prediction all-cause mortality outcome, NHANES 1999-2002 (n=2,402). Bold indicates statistical significance (p-value<0.05), and bold with two asterisks indicates significance passed Bonferroni adjustment (p-value<0.0021).

	Overall sample (N=2,042)	
	**Beta**	**p-value**
**Chronological age clocks**		
Hannum	-5.38E-04	0.174
Hovarth	9.51E-05	0.825
Weidner	2.75E-04	0.126
Lin	4.44E-05	0.737
Vidal-Bralo	3.97E-04	0.223
SkinBlood	2.79E-04	0.519
Zhang	2.85E-05	0.992
**Physiological functioning clocks**		
PhenoAge	4.09E-05	0.911
GrimAge	-7.31E-04	0.283
GrimAge2	-7.38E-04	0.305
**Pace of aging clock**		
DunedinPoAm	-3.21E+00	0.260
**Clocks trained on other aging biomarkers**		
Yang	5.17E+00	0.931
Telomere	-3.54E-01	0.317
**Behavioral/Lifestyle measures**		
Sedentary behavior	NA	NA
Healthy eating index	7.68E-05	0.629
Smoking pack years	-3.75E-05	0.149
Alcohol drinker	NA	NA
**Anthropometric measures**		
Waist to thigh ratio	1.57E-01	0.769
BMI	**3.35E-03****	**0.000**
**Clinical used blood Biomarkers**		
Total cholesterol	**6.61E-02****	**0.001**
HDL	**2.24E-04**	**0.015**
LDL	3.57E-05	0.057
Glucose	2.86E-06	0.523
C-reactive protein	**-3.69E-02****	**0.002**
