# Supplementary material for: The mediating role of DNA methylation clocks in associations of race, ethnicity, education, income, and occupation with mortality: findings from NHANES 1999-2002
Source: Aging (Albany NY). 2026 May 8;18(1):466–512. doi: 10.18632/aging.206377 (PMC13632862; doi:10.18632/aging.206377)
Supplement: Supplementary file 10 [file aging-18-1-206377-s001.docx]

**Supplementary Material**

**The Mediating Role of DNA Methylation Clocks in Associations of Race, Ethnicity, Education, Income, and Occupation with Mortality: Findings from NHANES 1999-2002**

Hanyang Shen, Nicole Gladish, Tiffany Fan, Andres Cardenas, Belinda L. Needham, David H. Rehkopf

**Table of contents**

Appendix 1

Appendix 2

**Appendix 1: Understanding the possible range of a mediated proportion**

The R *mediation* package can potentially output a mediated proportion ranged from negative infinity to positive infinity [1]. Instead of indicating mathematically instability or scaling artifacts, big proportions of mediation indicate that mediators (DNA methylation clocks) are good biomarkers to capture the effects on the outcome (risks of mortality) caused by the exposure (social positions). Here, we discuss the meaning of different possibilities using a simplified mathematical illustration:

Assuming the true model between exposure and outcome is

$y= \beta_{1}exposure+\varepsilon$ ,

when we add a mediator in the model

$y= \tilde{\beta}_{1}exposure+\beta_{2}mediator+u$.

Then $\tilde{\beta}_{1}= \beta_{1}-\beta_{2}\frac{Cov (exposure, mediator)}{Var(exposure)}$

The proportion of mediation (PM) is defined as

$PM=\frac{({\beta_{1}-\tilde{\beta}}_{1})}{\beta_{1}}= \frac{\beta_{2}}{\beta_{1}} \frac{Cov (exposure, mediator)}{Var(exposure)}$=1-$\frac{\tilde{\beta}_{1}}{\beta_{1}}$

Note that it is undefined if $\beta_{1}=0.$

Assuming the variance of exposure is a constant, $\beta_{1}and \beta_{2}$have the same sign (which means the association between outcome and exposure and the association between outcome and mediator are either both positive or both negative):

1) **A mediated proportion of 0**. $PM=0.$ This occurs when$\beta_{2}=0$or $Cov(exposure, mediator)=0$ , i.e., clinically either (i) the mediator has no association with the outcome conditional on exposure, or (ii) exposure and mediator are uncorrelated.
2) **A negative mediated proportion**. $PM<0.$ This occurs when $\frac{\tilde{\beta}_{1}}{\beta_{1}}>1$, meaning $\tilde{\beta}_{1}$has the same sign as $\beta_{1}$but larger magnitude. Adjusting for mediator strengthens the exposure coefficient. Under our “same sign” assumption, this is equivalent to $Cov\left( exposure, mediator \right)<0$. Clinically, this is a suppression/inconsistent mediation pattern: the exposure is negatively associated with the mediator, while both exposure and mediator are associated with higher risk (or both with lower risk).
3) **A mediated proportion larger than 100%.** $PM>1.$This occurs when $\frac{\tilde{\beta}_{1}}{\beta_{1}}<0,$ meaning $\tilde{\beta}_{1}$ and $\beta_{1}$have opposite signs. The introduced change $\beta_{2}\frac{Cov (exposure, mediator)}{Var(exposure)}$ is large enough to flip the sign of the exposure coefficient. Clinically, this indicates cancellation between “direct” and “indirect” components: the pathway through mediator and the remaining direct association act in opposite directions, so the inferred mediated component can exceed the total.
4) **An empirical example from our unpublished data**. Below is the mediation analysis for the association between being a smoker and all-cause mortality using the same method from our main analyses. As suggested in the literatures, smoking can undoubtedly cause adverse effects on health but can also be associated with stronger close social network which may be protective for adverse health effects [2]. The results below indicate that GrimAge and GrimAge2, containing a component specifically designed for smoking pack-years, capture the adverse effects of being a smoker bigger than the raw effect of being a smoker (indicated as PM>100%). Also the Weidner epigenetic age showing a significant negative proportion of mediation supports the protective effect of being a smoker through social support, since one of our previous studies reported Weidner epigenetic age is related to the pathway of social support affecting all-cause mortality [3].

In addition, change on the scale of the mediator did not change this result. We did further sensitivity analysis using z-score standardized measures of the DNA methylation clocks, and the results stay the same. This also makes sense mathematically since when reduced the scale of the mediator, the covariance reduced but the $\beta_{2}$ relatively increased.

**Appendix 2: Assumptions for the *mediation* package**

The mediation package holds a couple of assumptions including 1) no unobserved pre-treatment confounding between social positions and mortality outcomes besides the covariates already controlled; 2) no unobserved pre-treatment and post-treatment confounding for the associations of DNA methylation clocks and mortality outcomes; 3) common support areas for social positions and DNA methylation clocks. The estimates from this causal mediation model would be biased when these assumptions were not met. We discussed to what extent these assumptions are believed to hold in this study below.

1) **Pre-treatment confounding.** Social positions have been considered as the fundamental cause of health in previous literatures [4], particularly for race and ethnicity which people were assigned before they were born. Most of the health behaviors, life experiences, and comorbidities are fundamentally affected by these factors. In addition, in this study we would like to consider earlier life variables, such as earlier childhood abuse, as those may happen before education attainment, longest held occupation, and household income as precursors rather than confounders, because some adverse effects of earlier childhood experiences would go through the pathways of these social positions [5]. Therefore, we presented the estimates without controlling for any earlier life variables in the study. We also agree that there are variables, such as genetic nurture that we did not measure in this study. Such variables may still confound the relationship between exposure and outcome, and this assumption is not completely meet.
2) **Post-treatment confounding.** We assumed in this study that most of the confounders for mediator-outcome association are on the causal pathway of exposure to mediators. As indicated by a previous Mendelian randomization study, we here describe that epigenetic aging is primarily downstream of lifestyle measures, rather than vice visa [6]. Controlling for variables of unhealthy lifestyle and chronic comorbidity would exclude the pathways of these factors affecting outcomes through mediators. However, because there is still some uncertainty around this assumption, and it is not universal in the literature, we also presented results of mediation effects of variables including potential unhealthy lifestyle and physical measures collected at the same time as DNA methylation clocks for comparison. Our results indicate the DNA methylation clocks have mediated a greater proportion of social mortality inequality as compared to traditional top clinical measures.
3) **Common support and positivity.** In our dataset, individuals could possibly be exposed to any combination of exposures and mediators at any level of the confounders (e.g. age, gender, and nativity). In order to validate this assumption, we made scatter plots between exposures and mediators across different levels of confounders, which showed a large amount of overlapping between exposures and mediators. For example, below is one of the scatter plots between black comparing to white and GrimAge2. We are not providing

all these scatter plots in our supplementary materials since there are more than 100 of them, and researchers interested in these plots can use our code on GitHub to regenerate these plots.

**References**

1.	Tingley D, Yamamoto T, Hirose K, Keele L, Imai K, Yamamoto MT. Package ‘mediation.’ Comput Softw Man. 2019; : 175–84.

2.	Christakis NA, Fowler JH. The collective dynamics of smoking in a large social network. N Engl J Med. Mass Medical Soc; 2008; 358: 2249–58.

3.	Shen H, Gladish N, Cardenas A, Needham BL, Rehkopf DH. Social support and epigenetic aging at the intersections of race, ethnicity, and gender: findings from NHANES 1999-2002. SSM-Popul Health. Elsevier; 2025; : 101892.

4.	Phelan JC, Link BG, Tehranifar P. Social conditions as fundamental causes of health inequalities: theory, evidence, and policy implications. J Health Soc Behav. Sage Publications Sage CA: Los Angeles, CA; 2010; 51: S28–40.

5.	Houtepen LC, Heron J, Suderman MJ, Fraser A, Chittleborough CR, Howe LD. Associations of adverse childhood experiences with educational attainment and adolescent health and the role of family and socioeconomic factors: a prospective cohort study in the UK. PLoS Med. Public Library of Science San Francisco, CA USA; 2020; 17: e1003031.

6.	McCartney DL, Min JL, Richmond RC, Lu AT, Sobczyk MK, Davies G, Broer L, Guo X, Jeong A, Jung J, others. Genome-wide association studies identify 137 genetic loci for DNA methylation biomarkers of aging. Genome Biol. Springer; 2021; 22: 194.
